# Positioning Methods and the Use of Location and Activity Data in Forests

**DOI:** 10.3390/f10050458

**Published:** 2019

**Authors:** Robert F. Keefe, Ann M. Wempe, Ryer M. Becker, Eloise G. Zimbelman, Emily S. Nagler, Sophie L. Gilbert, Christopher C. Caudill

**Affiliations:** 1 Department of Forest, Rangeland and Fire Sciences, University of Idaho, 875 Perimeter Drive, Moscow, ID 83844, USA; 2 Department of Fish and Wildlife Sciences, University of Idaho, 875 Perimeter Drive, Moscow, ID 83844, USA

**Keywords:** Global Positioning System, Global Navigation Satellite System, radio frequency identification, ultra-wideband, radio telemetry, passive integrated transponder, big data, Internet of Things, location-based services, activity recognition, wearable technology, mesh network, geofence, forestry, wildland fire, fisheries, wildlife

## Abstract

In this paper, we provide an overview of positioning systems for moving resources in forest and fire management and review the related literature. Emphasis is placed on the accuracy and range of different localization and location-sharing methods, particularly in forested environments and in the absence of conventional cellular or internet connectivity. We then conduct a second review of literature and concepts related to several emerging, broad themes in data science, including the terms *location-based services (LBS)*, *geofences*, *wearable technology*, *activity recognition*, *mesh networking*, *the Internet of Things (IoT)*, and *big data*. Our objective in this second review is to inform how these broader concepts, with implications for networking and analytics, may help to advance natural resource management and science in the future. Based on methods, themes, and concepts that arose in our systematic reviews, we then augmented the paper with additional literature from wildlife and fisheries management, as well as concepts from video object detection, relative positioning, and inventory-tracking that are also used as forms of localization. Based on our reviews of positioning technologies and emerging data science themes, we present a hierarchical model for collecting and sharing data in forest and fire management, and more broadly in the field of natural resources. The model reflects tradeoffs in range and bandwidth when recording, processing, and communicating large quantities of data in time and space to support resource management, science, and public safety in remote areas. In the hierarchical approach, wearable devices and other sensors typically transmit data at short distances using Bluetooth, Bluetooth Low Energy (BLE), or ANT wireless, and smartphones and tablets serve as intermediate data collection and processing hubs for information that can be subsequently transmitted using radio networking systems or satellite communication. Data with greater spatial and temporal complexity is typically processed incrementally at lower tiers, then fused and summarized at higher levels of incident command or resource management. Lastly, we outline several priority areas for future research to advance big data analytics in natural resources.

## 1. Introduction

In this paper, we describe and review literature on several technologies and estimation methods that can be used to determine and communicate the position, navigation, and timing (PNT) of moving objects, equipment, people, fish and wildlife, and even physical masses (e.g., air, water, fire) in forests. We initially conducted a systematic review of the first twenty Google Scholar search results for each positioning system term, followed by the words forest and wildland fire, both singly and paired with accuracy and range, as documented in Appendix A. Technological terms searched included GPS (Global Positioning System), GNSS (Global Navigation Satellite System), Bluetooth, UWB (ultra-wideband), INS (inertial navigation system), RFID (radio frequency identification), and QR (quick response) code. Technologies described in this paper employ various positioning methods. Many make use of one or more GNSS constellations. Although GNSS has been used widely in natural resources for decades, accuracy continues to improve as the number of navigation satellites increases and more devices are designed to receive signals from multiple constellations [1]. Other positioning methods use trilateration of distances derived from the strength or timing of transmitted radio signals (e.g., RFID, UWB) or employ the inertial sensors in smartphones or watches to determine position as a function of the distance and direction traveled from a known reference point (e.g., INS) [2]. In inventory and asset tracking applications, the location and timing of resources at checkpoints along a supply chain can be scanned using RFID tags, QR codes, or other near-range mobile scanning methods; thus, RFID tags and QR codes are also forms of positioning [3–5]. Additionally, in many cases, communication technologies such as Bluetooth, very high frequency (VHF), UWB, and other radio frequencies or protocols may be used to (1) determine the location of an individual or object through trilateration; (2) transmit positions or other data among devices such as a fitness watch sending data to a smartphone via Bluetooth Low Energy (BLE) or ANT wireless; or (3) both 1 and 2 simultaneously. After reviewing and summarizing search results from our initial systematic review of positioning systems, we supplemented preliminary materials with related literature from radio and acoustic telemetry in wildlife and fisheries management and added the concepts of video object detection, optical positioning, and relative positioning in reference to drones and equipment automation.

Improvements in the accuracy of affordable, GNSS-based devices combined with the emergence of other localization techniques are enhancing our ability to map people, animals, equipment, and other resources at high spatial and temporal resolutions. For example, integration of multiple positioning methods such as a combination of GNSS and INS, can increase positioning accuracy under forest canopies [6,7]. Additionally, with developments in off-the-grid communications for areas that lack conventional cellular data access, we can share real-time position information in forests and other remote environments using networked two-way radios and direct satellite data transfer.

After reviewing common positioning and location- or data-sharing methods, we conducted a second review of several broad data science concepts that may influence natural resources in the future. We considered the most relevant of the first twenty results in our initial searches, and then expanded the review to additional, related literature that helped broaden or clarify themes. Search terms initially included *location-based services (LBS)*, *geofences*, *wearable technology*, *activity recognition*, *mesh networking*, *the Internet of Things (IoT)*, *and big data*. *Location-based services* are algorithms, apps, or other networked processes that perform a specific function based on the current location or heading of a user. Similarly, *geofences* quantify, inform, or influence resource movements or positions based on real-time coordinates. *Activity recognition* refers to the growing use of *wearable technology* such as fitness bands, GNSS-enabled smartwatches, and other devices, to self-monitor personal movement and physiological status [8–10]. Common smartphones, equipped with a range of inertial sensors, can also be used for activity recognition [11–14]. The concept of the *quantified self* [15], in which individuals are empowered to study their lifestyles and behaviors with increasingly available data, is widespread in fitness, recreation, and sports, and activity monitoring is quickly moving into the workplace to increase employee wellness, reduce corporate healthcare costs, and improve productivity [16–18]. Parallel developments in animal telemetry are generating detailed data on animal behavior based on accelerometer and inertial sensor data capturing movement and activity [19].

There are several possibilities for sharing the kinds of high-resolution data that are created by activity recognition and other mobile device applications. For example, using either advanced digital radios or miniaturized antennas that pair with smartphones via Bluetooth or BLE, users can share location and other types of data through local *mesh networks*, even in remote, off-the-grid areas lacking cellular or Internet services [20,21]. Alternatively, devices that use satellite data networks for two-way communication have global connectivity. There are tradeoffs, however, among available bandwidth and desired effective transmission range spanning from adjacent (1 m), to watershed (e.g., 500 m), or global spatial scales. In natural resources, smartphones and tablets increasingly serve as the local processing units. For *big data* applications, data can be transmitted via radio or satellite to off-site computers that extract useful spatial and temporal information in higher order analytics. Smartphones and other devices are becoming more interconnected in remote areas as new networking capabilities bring the concept of an *IoT* to the woods.

Emerging technologies that enable enhanced localization and data transfer in off-the-grid applications evoke research, operational, and analytical challenges [21–23]. For example, characterizing the activities of multiple moving resources (e.g., 1000 or more people and equipment on a wildland fire) at various scales in real-time or near real-time requires overcoming obstacles associated with available bandwidth, power consumption, and signal transmission quality and interval. There is growing disparity between (a) increasingly high bandwidth networking capability intended to support smart devices and IoT in more suburban areas, (b) transitional zones of sparser or more sporadic connectivity at the wildland urban interface (WUI), and (c) very poor network capability in more remote areas. Thus, diverse and variable communications infrastructure may be needed in the future to support analytics. Based on our review of available positioning methods and emerging big data concepts in location-based services, wearable technology, and the IoT, we propose a simple, general approach for multi-tiered data collection, incremental processing and analysis, and network communication in forest management and wildland fire. We feel this concept provides a general, interim solution for scalable data collection and analysis off-the-grid that can be deployed readily using available tools and technology. It is likely of equal value and applicability in most natural resource fields, including, but not limited to, recreation, wildlife and fisheries management, range ecology and management, and research and monitoring. We also identify nine priority research needs to advance basic and applied science in this broad subject area.

## 2. Types of Positioning Technologies

### 2.1. GNSS – Single, Dedicated Receiver

The Navigation System with Timing and Ranging (NAVSTAR) GPS was developed by the United States (U.S.) Department of Defense during the early 1970s for military applications as well as for civilian use [24]. The term GNSS refers broadly to the American GPS system and other satellite-based navigation constellations developed subsequently in other regions to enhance terrestrial and maritime PNT for various fields [2]. The other three primary systems currently in use and continued development are the Russian Global Navigation Satellite System (GLONASS), the European Union’s Galileo system, and the BeiDou-2 system developed by the People’s Republic of China. While GPS and GLONASS have been in use for some time, Galileo first became operational in December 2016, and BeiDou-2 is expected to have operational global coverage in 2020 [1,25]. This is of interest because improved positioning accuracy is possible with integrated multi-GNSS frameworks that incorporate all four GNSS systems as the total number of available satellites across all constellations increases from 70 to approximately 120 over a period of a few years [1].

**Figure 1.**
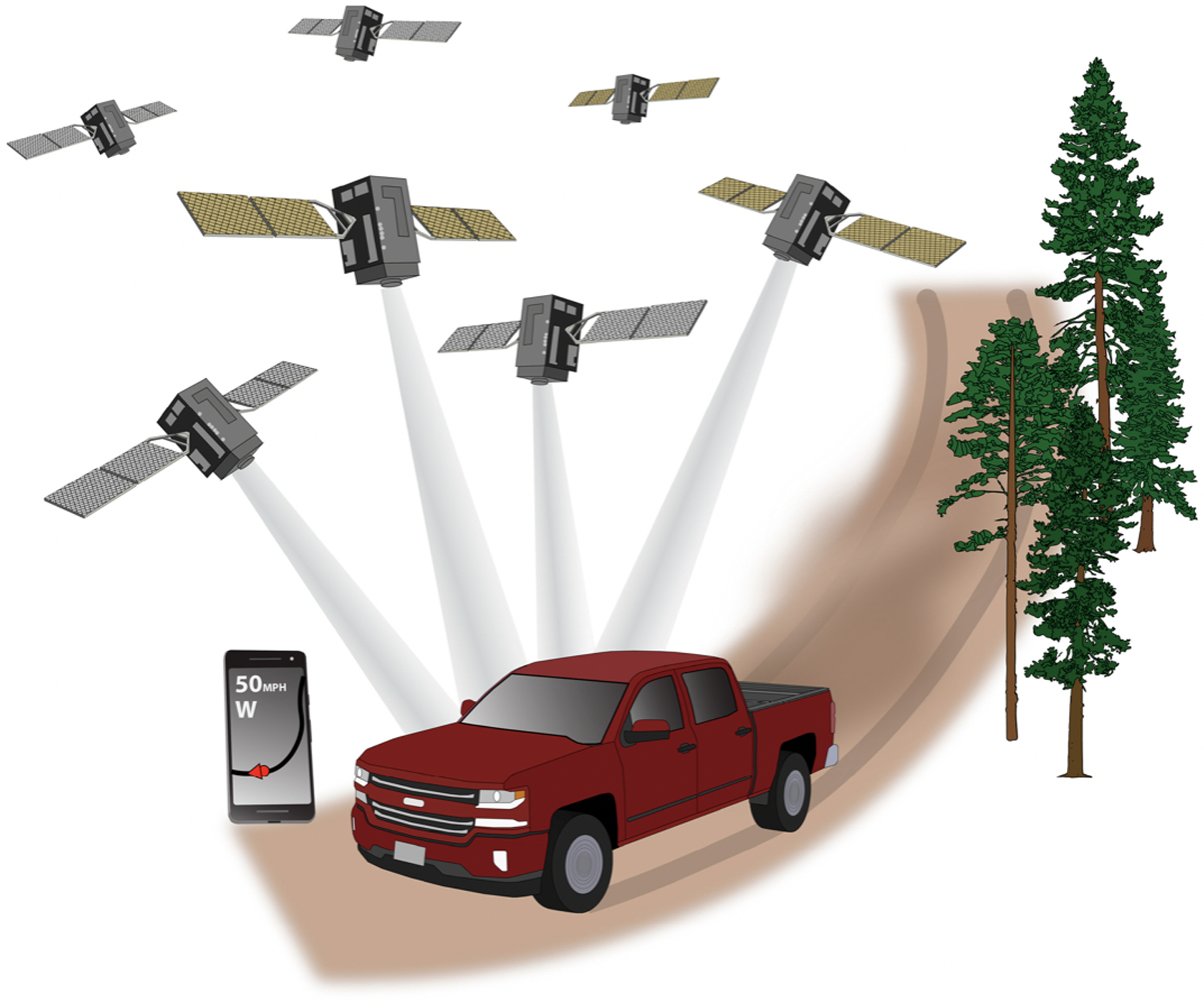
The Global Positioning System (GPS) and, more broadly, the Global Navigation Satellite System (GNSS), rely on three or more navigation satellites to determine ground position of receivers. Position is determined based on the time-delay associated with signal reception relative to synchronized clocks on each satellite. GNSS devices with the capacity to integrate signals from two or more international constellations are increasingly available. The rapidly growing number of satellites generally corresponds with increasing accuracy in a wider range of devices over a 5–10 year transition period.

Figure 1 depicts the GNSS receiver chip in a smartphone being used for vehicular navigation. The mobile device receives timing and range data from several satellites and subsequently uses these to determine its location on the Earth’s surface. The single device user can use positioning information such as latitude, longitude, elevation, and vector heading to monitor his or her location on topographic maps, custom maps, and aerial imagery. Prior to 2000, selective availability (SA), an intentional degradation of GPS signal quality by the U.S. government for national security reduced the accuracy of satellite positioning in the U.S. [26]. Adrados et al. demonstrated the significant improvement to accuracy with the removal of SA in May 2000, with mean error for a survey-grade receiver decreasing from 30 meters (m) to less than 2 m in ideal conditions [27].

Of the three general categories of GNSS receivers (survey-, mapping-, and consumer-grade), survey-grade receivers are the most accurate, able to achieve positioning accuracies of 1 m or less under forest canopies and approximately 5 centimeters (cm) in open areas (see Table 1) [28,29]. For survey-grade devices, accuracy increases with longer durations of continuous position logging [30]. Typical accuracy of consumer-grade GNSS in U.S. forests ranges from 1 to 5 m in open conditions [28,29,31–33] and from 3.8 to 12 m under mature canopies [28,29,31–34].

The effect of canopy on GNSS signal transmission has been well-documented, with accuracy inversely proportional to stand density in most situations [21,23,29,31,33–36]. GNSS accuracy does not vary by the time of year (season) or weather [34], but can be affected by device orientation when held [37].

The number and geometry of satellites visible to a GNSS receiver at any point in time also affect the accuracy of coordinate readings recorded by the device. Various dilution of precision (DOP) indices are used to characterize the influence of satellite geometry on GNSS measurements [38]. Positional DOP (PDOP) is a particularly common index of the uncertainty in overall position, with lower PDOP values indicating a satellite arrangement providing higher measurement accuracy and precision [38].

**Figure 2.**
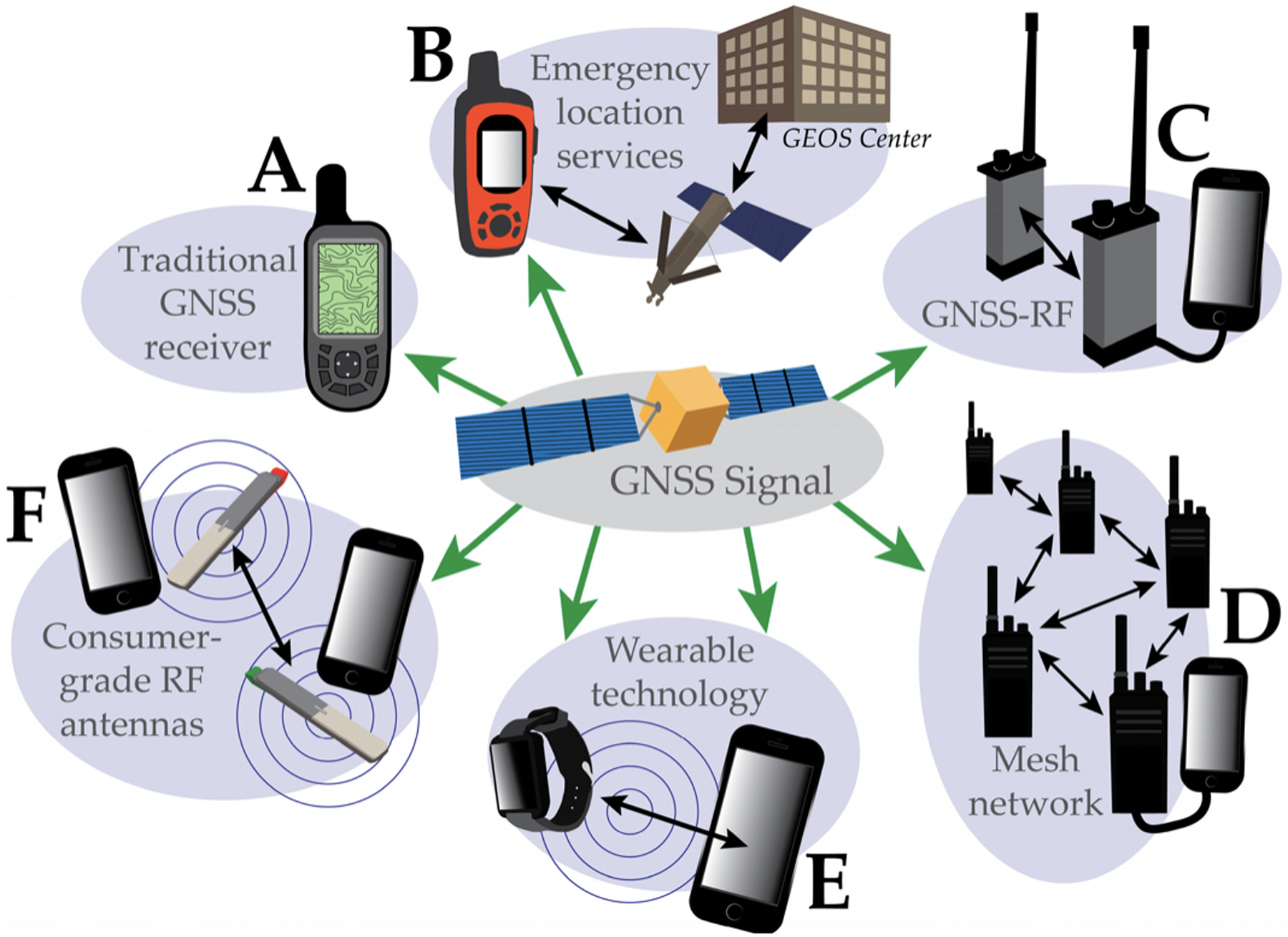
Common types of available technologies for real-time location sharing in remote areas. A. Conventional handheld GNSS receiver displays the user’s location. B. Two-way satellite communication using Iridium or other commercial satellite communication networks make it possible to send emergency SOS signals or text messages relayed through the communications satellite. C. GNSS transponders paired with radio frequency (RF) transmission (GNSS-RF) send locations between devices, so that location coordinates can be mapped on phones or tablets connected to receiving devices that are within radio line-of-sight. D. Advanced data radios with mesh networking function the same as (C), but in this case each radio (node) serves as a repeater, improving network connectivity around topographic or other obstacles in remote areas. E. GNSS-enabled watches transmit user location, inertial sensor and other sensor data (e.g., heart rate) to the user’s phone using Bluetooth, Bluetooth Low Energy (BLE) or ANT wireless. F. Miniaturized radios that pair with smartphones using Bluetooth or BLE provide the same capabilities as (D) but utilize the existing GNSS chip in phones providing location sharing functionality at lower costs than dedicated radios.

Individual GNSS receivers (Figure 2, panel A) have been commonplace in forestry and throughout natural resource science and management for decades. In forestry and wildland fire, handheld GNSS receivers have been used for a wide variety of geospatial mapping and navigation applications. These include navigation to forest inventory plots and monumentation [39], as well as delineating stand boundaries and other features on stand maps [40,41]. Handheld receivers have also been used to a limited extent to quantify the productive cycle times of logging equipment [42–45] and soil impacts of harvesting equipment [46,47]. Use of single-unit GNSS to assist with navigation and to record travel paths for heavy equipment in forestry is now fairly common [30,48–50]. Onboard computers and software sold by large equipment producers such as Caterpillar, John Deere, and Ponsse have built-in GNSS receivers with the ability to map equipment locations on harvest maps, showing machinery proximity to unit boundaries or streamside management zones [20].

Single-user devices have also been used for personal location, safety, and field navigation in the woods by foresters and other natural resource managers for decades. Handheld Garmin, Trimble, or other consumer-grade GNSS units have traditionally been a standard tool attached to the field vest of most foresters when they head to the woods. GNSS accuracy provided improved estimation of land area over traditional compass and pace field methods [51]. Individual GNSS receivers have become increasingly small in stature over time; what were originally backpack-worn units are now small chips that fit inside smartphones and smartwatches [29,52]. As in forestry, use of handheld GNSS receivers has been commonplace in wildland and prescribed fire for maintaining reference position, marking waypoints of snags or other hazards, relaying coordinates of spot fires and delineating burn perimeters to determine area burned [53]. Multi-temporal burn perimeter data combined with current wind speed and direction can also provide inputs for forecasting predicted fire spread [54].

### 2.2. GNSS—Smartphone and Tablet-Based Mapping

While the dedicated GNSS devices described in the previous section were ubiquitous in natural resources until recent years, positioning via the GNSS chip present in most modern mobile devices and tablets is now increasingly common [55–57]. Mobile-based apps such as Avenza Maps^®^ store georeferenced, custom maps in GeoTIFF or GeoPDF format on mobile devices, allowing users to view positions on a smartphone or tablet rather than on a conventional, dedicated GNSS unit [58,59]. The United States Department of Agriculture (USDA) Forest Service, United States Geological Survey (USGS), and many other federal and state agencies, as well as private forest industry landowners, increasingly make GeoPDF map files available to the public or contractors for use with mobile-based mapping apps [60]. These applications rely only on the satellite-derived GNSS coordinates received by the device and display the user’s location on a locally-saved version of a map file [61]. They therefore require neither cellular service connectivity nor WiFi for mapping and are thus fully functional in remote areas given map files are downloaded to the device in advance. This approach of alternating between online and offline data collection paired with GNSS-based location information on smartphones and tablets has been used widely in operational forestry and more recently in research applications such as mapping beetle outbreaks [62] and landslide hazard mapping [57].

GNSS accuracy of modern smartphones is approximately 2 m in open conditions and ranges from about 4.5 to 6.7 m (leaf-off) and 6.7 to 11.5 m (leaf-on) in mixed deciduous-coniferous forests [29]. Accuracy varies by brand and model, but devices supporting both GPS and GLONASS are generally able to achieve higher accuracies under forest canopies [29].

### 2.3. Augmented GNSS and GNSS with RTK Correction

**Figure 3.**
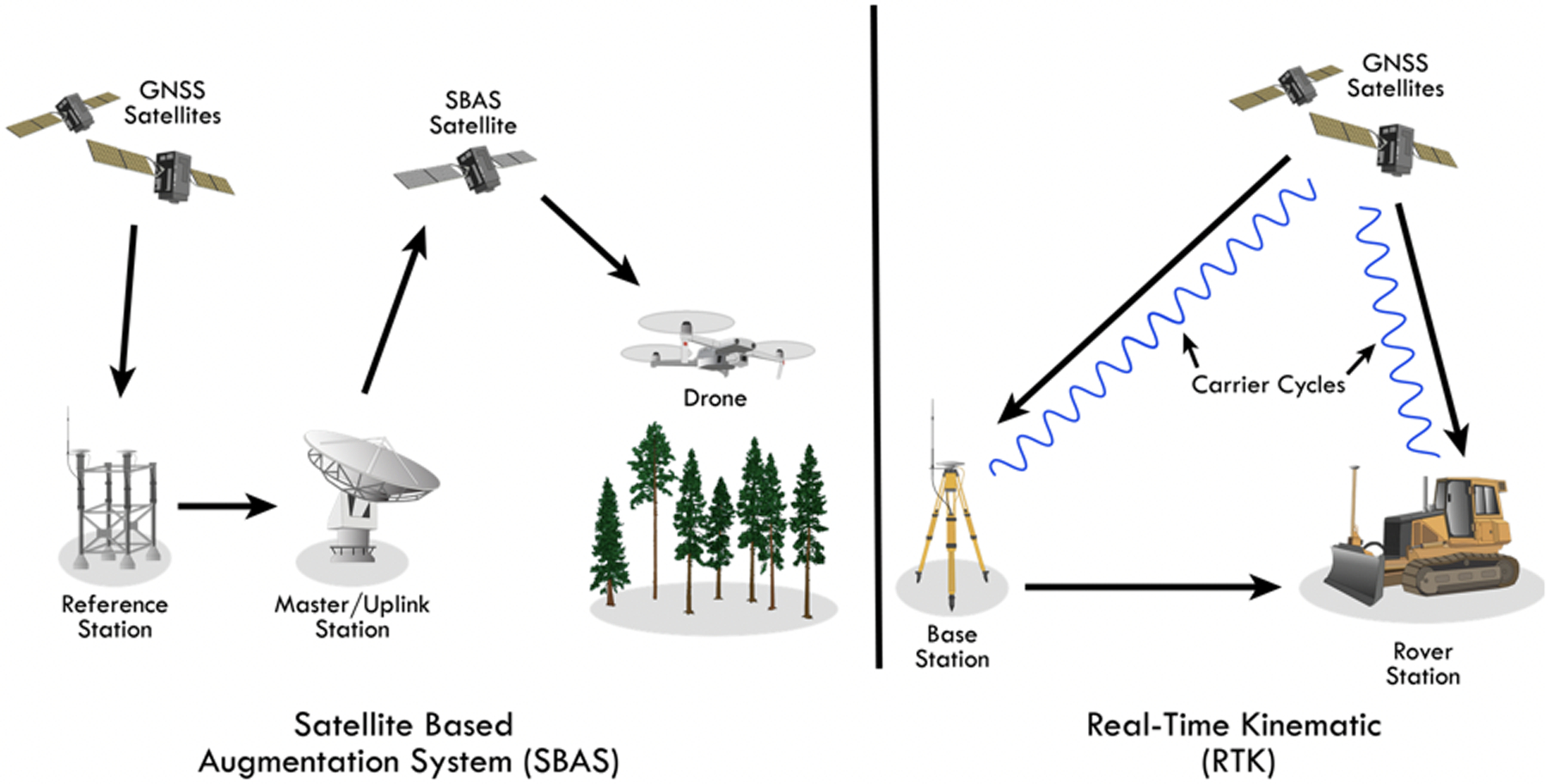
In a Satellite-Based Augmentation System (SBAS) (**left** panel), reference stations located at precisely-surveyed points receive and forward their GNSS signals to the master station, which then calculates corrections. These corrections are sent to SBAS satellites, which in turn broadcast correction messages to the end user. In a Ground-Based Augmentation System (GBAS), such as real-time kinematic (RTK) positioning (**right** panel), a base (reference) station at a known location and a mobile rover both receive pseudorange and carrier phase measurements from similar satellites. Common errors between the units are estimated and correction data from the base station is transmitted to the rover for use in real-time or is utilized in post processing.

With either dedicated, single unit GNSS receivers or mobile device-based GNSS solutions, several methods exist to improve upon the accuracy of GNSS positioning. One such method is a satellite-based augmentation system (SBAS), in which reference stations located at known points are used to create correction messages that are sent to various satellites and then broadcast to end users (Figure 3, left panel) [63]. The Wide Area Augmentation System (WAAS) in the United States, which is managed by the Federal Aviation Authority (FAA), is an example of an SBAS [64]. The WAAS encompasses a network of terrestrial reference stations that estimate errors in GPS signals and send correction messages to a geostationary communications satellite (GEO) which then re-transmits the corrections to any WAAS-enabled GNSS device [65] as shown in Figure 3. WAAS differential correction is frequently built into GPS devices and even some smartwatches, but has demonstrated little positioning accuracy improvement for consumer-grade receivers [64]. Arnold and Zandbergen compared WAAS-enabled modes to autonomous (uncorrected) modes for Garmin, Trimble, and DeLorme receivers under ideal conditions; WAAS improved horizontal positioning accuracy only for the Delorme units [64]. Additionally, WAAS signaling is limited under forest canopy, typically available less than one quarter of the time for stationary, recreational-grade devices, and even less for mobile receivers [65].

In a ground-based augmentation system (GBAS), correction messages are transmitted to the end user via radio or mobile data network [63]. Real-time kinematic (RTK) positioning is a GBAS differential correction method in which one or more base (reference) stations at fixed, precisely-surveyed locations are used to improve the accuracy of mobile rover units [63,66,67]. Specifically, both the reference and rover units receive pseudorange and carrier phase measurements from similar satellites, which allows common errors between the units to be estimated and used to correct the rover’s accuracy in real time [66,67]. Real-time kinematic is typically built into survey-grade devices, enabling them to achieve centimeter-level accuracy in unobstructed conditions (see Table 1) [29] and is integrated into heavy equipment for road construction (Figure 3, right panel) [68,69]. Real-time kinematic methods improve accuracy under the canopy as well, but are not immune to multipath error inherent in forests; therefore, positioning accuracies are lower (about 1 m) in these highly reflective environments [29,63,66].

Precise point positioning (PPP) is an absolute positioning technique that uses a single GNSS receiver and corrections for satellite positions and clocks based on processing of reference station data available through the International GNSS Service (IGS) [70]. Receivers simultaneously use dual frequency pseudorange and carrier phase measurements in order to improve accuracy [67,70]. Data downloaded from the IGS can be used in real-time or for post-processing correction [63,71]. With PPP, GNSS devices can correctly position within 5 cm in open areas and less than 0.5 m under forest canopies (Table 1) [67,70].

### 2.4. GNSS with Two-Way Satellite Communication

Where forestry and wildland fire activities occur in proximity to the built environment, it is possible to relay location coordinates to others via mobile data transfer using conventional cellular communication and Internet connectivity. Google location services on Android-based mobile devices, for example, make it possible to share the location of one’s smartphone with other individuals over the Internet [72]. Positioning of devices in the built environment where cellular coverage is available can be done with high accuracy due to cellular tower trilateration, map-matching, and other methods integrated into phones or apps that improve on or substitute for GNSS-based positioning [73–77]. However, forestry and wildland fire management activities often occur in areas where cellular coverage is poor; thus, methods that rely on cellular connectivity are primarily useful in portions of the Wildland Urban Interface (WUI). Even in these areas, it is common for cellular connectivity to be lost or overloaded during large fires and other natural disasters [78].

Satellite communications technology allows for messaging and voice communications independent of cellular infrastructure [79,80]. Over the last decade, multiple devices have been marketed for forestry and recreation that provide emergency location services to individuals working or recreating in remote environments [20,80,81]. These include, for example, SPOT^™^ receivers and Garmin inReach^™^ two-way messaging devices (Figure 2, panel B). GNSS coordinates are transmitted along with automated or custom text messages to the urban environment via dedicated data communication satellites [80,81]. Both SPOT and inReach devices can send a user’s GNSS location, messages indicating safety status, and alerts to contact emergency services [81,82]. Depending on the model, certain SPOT receivers only allow for pre-loaded messages and one-way communication while other SPOT receivers and all inReach devices allow for both preset and custom messages as well as two-way messaging [82,83]. SPOT receivers operate on the Globalstar satellite network while inReach devices operate on the Iridium satellite network [82,83]. Iridium is being replaced with the Iridium NEXT constellation that will provide broader coverage and support more bandwidth and higher speeds [84]. Insofar as multiple individuals may carry devices (e.g., wildland fire crew, recreation crews, wildlife technicians), emergency location and messaging devices can form a simple multi-unit network. However, location information is primarily intended for outside supervisors or support resources in an urban environment elsewhere [80,81]. As of 2018, the frequency with which locations can be transmitted using paid-monthly services using Garmin inReach is one location every two minutes [82]. The fastest tracking interval for SPOT receivers at the time of preparing this manuscript is every 2.5 min [83]. Many consumer-grade GNSS transponder devices are available for less than US $100 to facilitate position observation independently from voice or text communication. However, for operational forestry and wildland fire applications, devices like inReach or SPOT receivers can be thought of as providing an open circuit rather than one-way location transmission.

### 2.5. GNSS-RF

**Figure 4.**
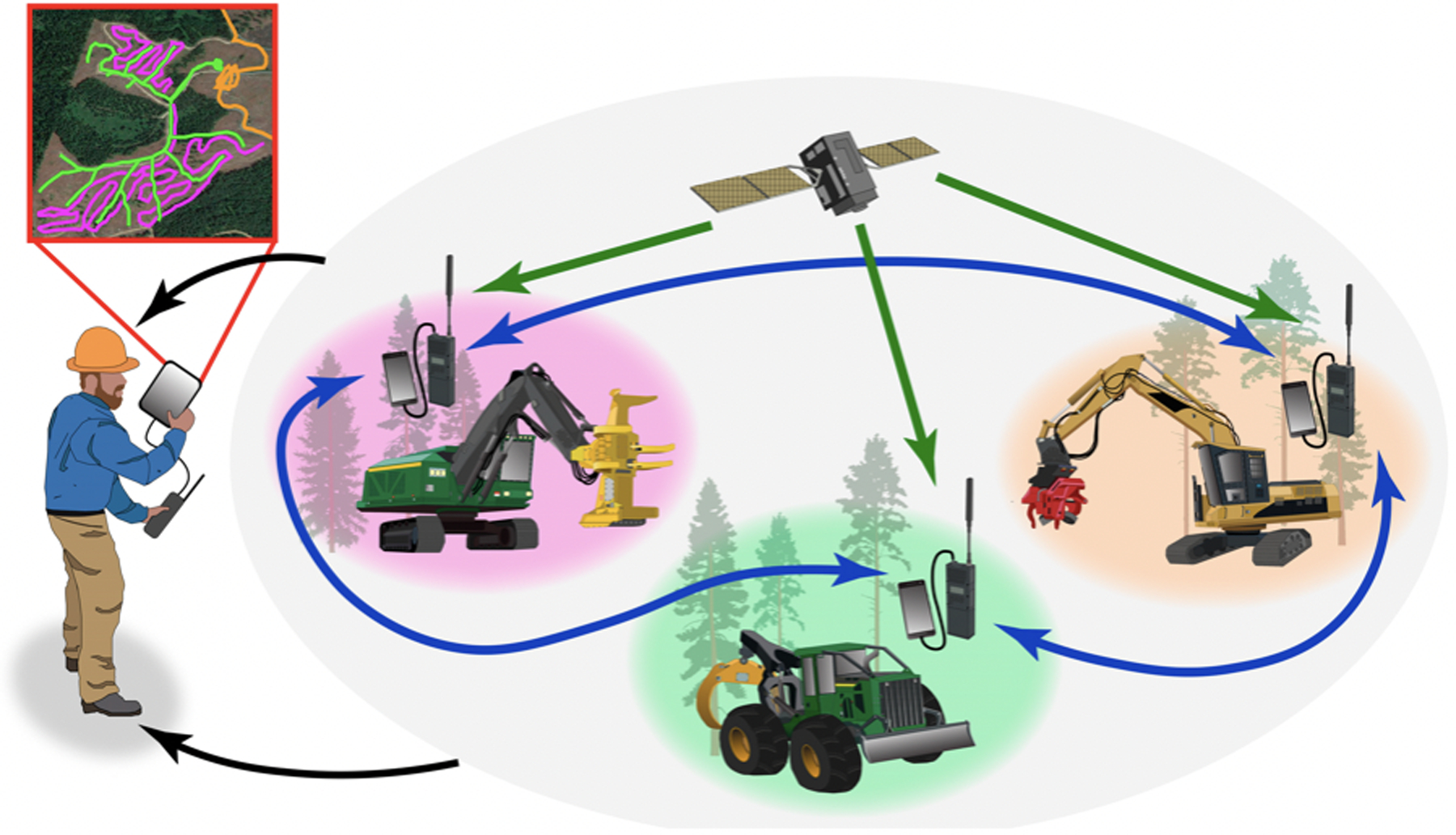
Example configuration for a real-time GNSS-RF network used in operational forestry to increase the safety and efficiency of logging operations. Each radio determines its coordinates using GNSS. Current positions of each piece of equipment, and possibly other data, are then transferred to other units via radio frequency at rates of typically one transmission per five seconds or less. One or more devices are attached (tethered) to tablets or other mobile devices to map the locations of all equipment in real time.

Individuals can share positioning information with one another locally through GNSS-RF networks (Figure 2, panel C), in which dedicated transponders or mobile devices determine coordinates using GNSS and then transmit those coordinates in short data bursts to other nearby devices via a radio frequency (RF) signal (Figure 4). GNSS-RF may be contained in a single unit, or GNSS-enabled devices like smartphones may be paired with miniaturized radios like goTenna^™^ or Beartooth^™^ to relay location information in areas lacking cellular coverage (Figure 2, panel F). This method has been evaluated widely for off-the-grid location sharing in operational forestry [20–22,85–88].

While quantifying the accuracy of single-user GNSS device applications described in previous sections is relatively simple, GNSS-RF and other systems that incorporate location sharing between multiple devices in remote areas pose different challenges for quantifying accuracy of position, navigation, timing, and other varieties of data among resources. This is due to the added impact of a temporal error component that sometimes interacts with spatial error and may be affected by other factors [21–23]. The temporal component becomes important in remote locations and data sharing because user positions and the associated data are (1) received or calculated on the target user’s device initially, and then (2) transmitted to other, neighboring devices. Most PNT research in natural resources has focused on error associated with the former component (target user location), and generally focused on static position accuracy. However, the latter process (regular transmission and sharing of locations from the primary device user to one or many neighbor devices) introduces time lag that is a function of both (a) the interval at which data packets are relayed between the devices and (b) transmission quality [21–23,85,86]. For example, the locations of the forestry equipment shown in Figure 4 may be received and determined at intervals of 100 hertz (Hz) on smartphones or GNSS receivers. However, those locations are transmitted to other devices at intervals that are typically 1 Hz, but possibly as infrequently as 2 Hz (one set of coordinates every 30 s) or less. Thus, even when radio transmission is clear, there is up to a 30 s gap that occurs when the location and vector heading of any worker or piece of equipment is unknown, from the perspective of those viewing activities on the mobile screen of a secondary device on the network. If a single transmission of coordinates (latitude, longitude), direction, and speed is missed due to topography or other interferences, that period of mapping uncertainty about the target user’s location and path increases to one minute, and so forth. This compounded error that includes spatial and temporal components is important for mission critical safety and health applications [21–23,87], particularly when we consider GNSS-RF as the basis for first responder life safety applications and heavy equipment automation.

### 2.6. Ultra Wideband and UHF/VHF Radio Telemetry

UWB is a radio frequency channel operating at bandwidths greater than 500 megahertz (MHz) that is primarily used for communications, wireless sensor networks, positioning, and tracking [89–91]. Wide bandwidths allow UWB signals to transmit large amounts of data quickly at low power consumption. Due to their low frequency, UWB signals can more easily pass through obstacles, making UWB well-suited for forested environments [90–92]. Additionally, using a bi-cone antenna system, UWB can provide communications in remote areas with a relatively low loss of data [90].

**Figure 5.**
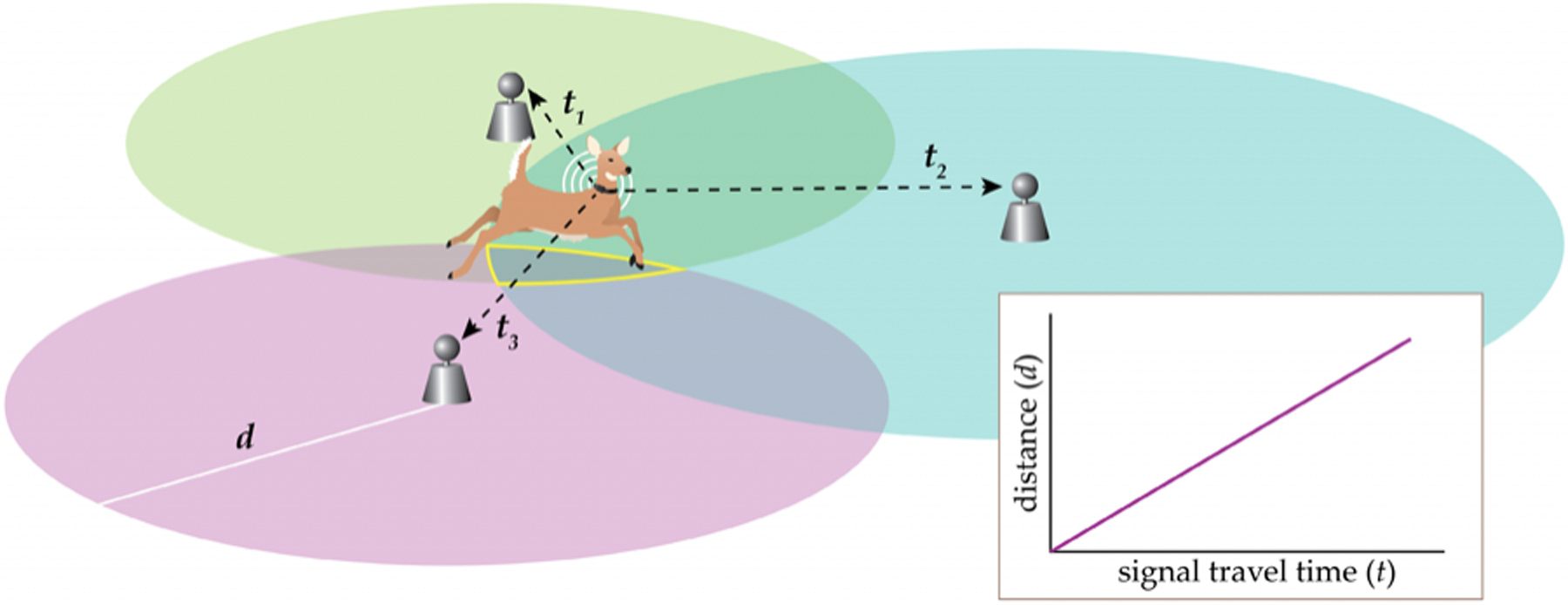
UWB positioning using TOA (time of arrival) trilateration. A tag (worn by the deer) emits UWB pulses which are received at each of the three anchor nodes. Based on a known signal speed and the propagation delay (signal travel time, *t*), each receiver can calculate its distance, *d*, from the tag. The area where the three distances intersect (shown with a yellow outline) is the estimated position of the tag [91,93].

Similar to GNSS, UWB positioning typically employs trilateration to estimate the location of a mobile tag (transmitter) based on the tag location relative to stationary anchor nodes (receivers) at known coordinates [91–93]. However, any of five general methods may be used for UWB positioning: angle of arrival (AOA), time of arrival (TOA), time difference of arrival (TDOA), received signal strength indication (RSSI), and hybrid algorithms [91,93]. In AOA, anchor nodes triangulate a tag’s position based on the direction of the received RF signal. For the time-stamp method of TOA, the anchor nodes use the propagation delay (signal travel time) and a known signal velocity to calculate their distances from the tag and then employ trilateration to estimate tag position (Figure 5). In another time-stamp method known as TDOA, anchors report signal receipt time to a location engine that compares the differences in arrival times and then estimates the location of the tag as the intersection of three hyperbolas (for two-dimensional positioning) [91,93]. To employ the RSSI method, a path loss regression model is first developed to establish distance as a function of signal strength [92]. Then, anchor nodes use received signal strength to predict distance and in turn estimate tag location with trilateration. This method has relatively low accuracy and is used less frequently than time and angle-based methods [91,92]. Lastly, hybrid algorithms combine methods to mitigate limitations of a single approach [91–93]. For example, AOA generally requires many more anchor nodes, while TDOA and TOA require precise synchronization among all anchor nodes. AOA and TDOA are frequently combined for improved positioning accuracy. UWB localization can typically achieve sub-meter accuracies (3–50 cm) even with consumer-grade units, though accuracy decreases in non-line-of-sight (NLOS) situations (see Table 1) [77,94]. Signals are capable of traveling long distances (approximately 100 m) with line-of-sight (LOS) conditions [95].

In wildlife ecology and management, animal tracking via technology attached to animals has improved dramatically in the past several decades and can now give researchers excellent spatiotemporal data on an ever-widening array of terrestrial and aquatic species (for two recent reviews, see Hussey et al. [96] and Kays et al. [97]). Traditional tracking approaches included (1) radio telemetry with VHF transmitters affixed to animals, which require manual or automated triangulation to record a location, or (2) pit tags and other passive location devices (e.g., geolocators for migratory birds [98]), in which locations are either recorded when animals approach powered antenna arrays or are stored on tags to be eventually recovered by researchers and downloaded. VHF and ultra high frequency (UHF) telemetry methods function essentially the same as UWB, and these frequencies have been used most commonly for research and management applications.

New wildlife monitoring technologies based on GNSS location are rapidly replacing older telemetry techniques for medium-sized animals and larger terrestrial animals (e.g., birds greater than 250 g and ground-dwelling fauna greater than 15 g [97]) due to rapid, ongoing advances in miniaturization. Designs include collars, ear-tags, backpacks, glue-ons that adhere to shells, scales, or fur, and internally-implanted models [97]. Positioning approaches for terrestrial wildlife usually involve GNSS devices that transmit information to researchers using commercially-available or private satellite networks, or cellular tower networks, and are thus functionally similar to safety devices such as Spot^™^ and Garmin inReach^™^ that also use two-way satellite data communication. For aquatic wildlife, GNSS loggers have also greatly improved researchers’ monitoring abilities, as has the advent of acoustic arrays that can monitor animals that never come to the surface [96]. Coupling these new location technologies with bio-loggers, which can measure animal physiological states (e.g., heart rate, caloric expenditure, and environmental conditions including sunlight, salinity, temperature, and depth), and even with drones equipped with onboard cameras and acoustic recorders, is leveraging these new technologies even further [98–100]. The resulting fine-grained and real-time data on animal locations and behaviors can be combined with monitoring systems to improve human and wildlife safety and to enhance conservation of rare species (e.g., proximity, immobility or death, geofencing, and movement or behavior classification [101]). One consideration that is important for active (i.e., UHF) radio tags and miniaturized GNSS transponders for wildlife, which may be less critical for operational forestry, is the need to conserve battery power. Receipt and transmission of coordinates from wildlife location devices must generally be limited to lower coordinate transmission intervals because of the potential for rapid loss of battery power, which is a lower concern for forest workers who can charge devices each evening, or onboard equipment applications with continuous power.

### 2.7. Inertial Navigation Systems

**Figure 6.**
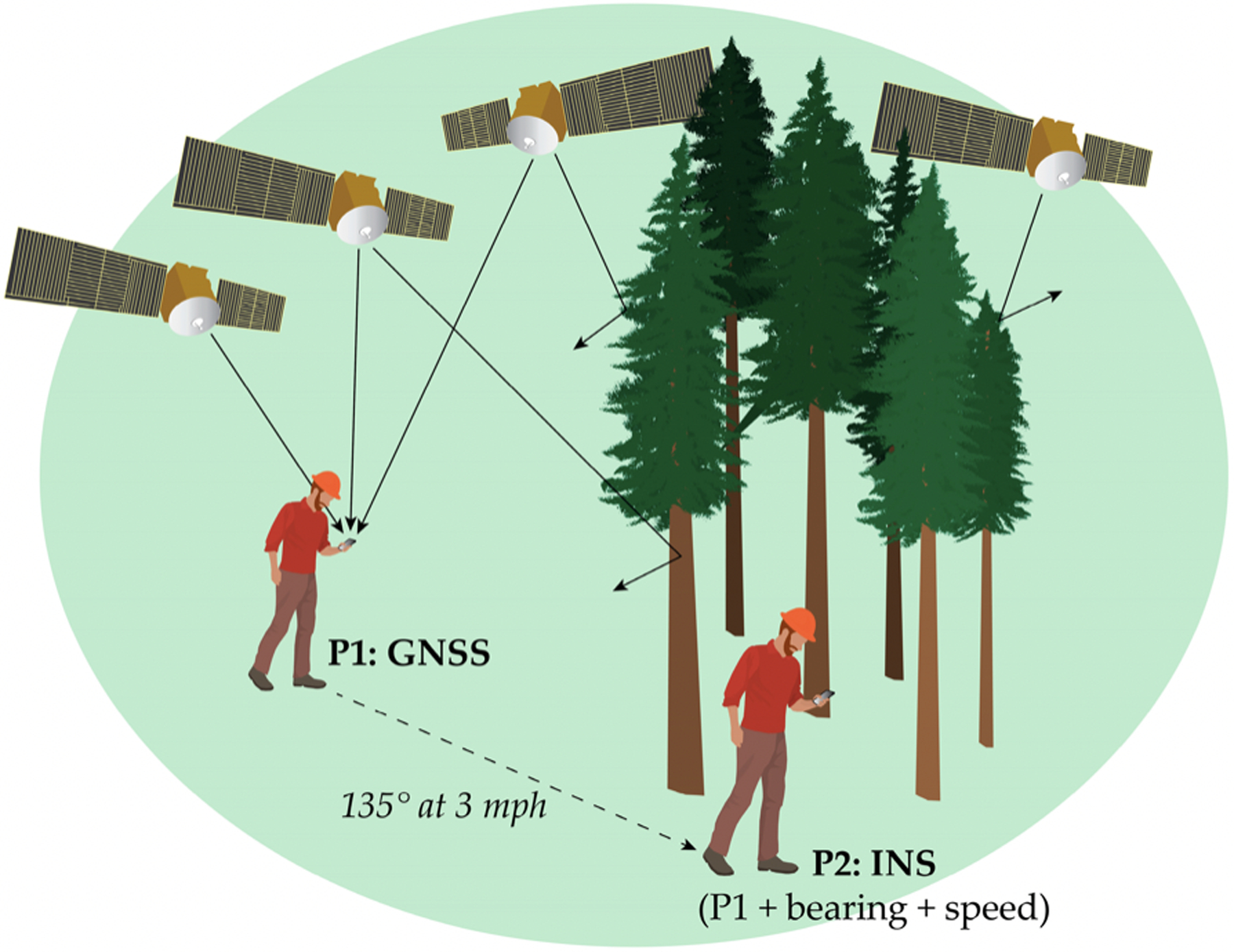
Inertial Navigation Systems (INS) in the forest. The forester’s coordinates are determined by GNSS at the first position (P1). The INS in his device tracks the direction and speed of his movement and can calculate his second position (P2) in the dense canopy based on where the forester has traveled from his last known GNSS coordinate.

INS are based on translational and rotational velocity, and how these two variables can be used in combination with GNSS to track and locate objects in the field [2]. GNSS signaling varies with topography, forest conditions, and other factors, but INS can fill in positioning information between known GNSS coordinates [2]. Using a combination of gyroscopes, accelerometers, and magnetic sensors, INS continually calculates mobile device location by dead reckoning. Dead reckoning is a navigation technique that estimates current position by summing sequential vector headings and estimated speed or distance from a known starting point, such as coordinates obtained initially from GNSS [102]. This approach is particularly useful in areas where individuals or equipment may move from areas with suitable GNSS accuracy to areas with degraded GNSS quality, such as traveling from a large meadow or clearcut into a dense forest stand (Figure 6). GNSS-INS accuracy varies with GNSS receiver quality, but these systems are generally capable of sub-meter positioning, with 4–5 cm possible using PPP (Table 1) [103–106]. Micro-electric mechanical systems (MEMS), which use miniaturized sensors, can reduce the size and cost of INS navigation, but MEMS accelerometers and gyroscopes generally exhibit higher errors than traditional INS. Calibration of these sensors is recommended for increased positioning accuracy [107].

### 2.8. Simultaneous Localization and Mapping (SLAM)

**Figure 7.**
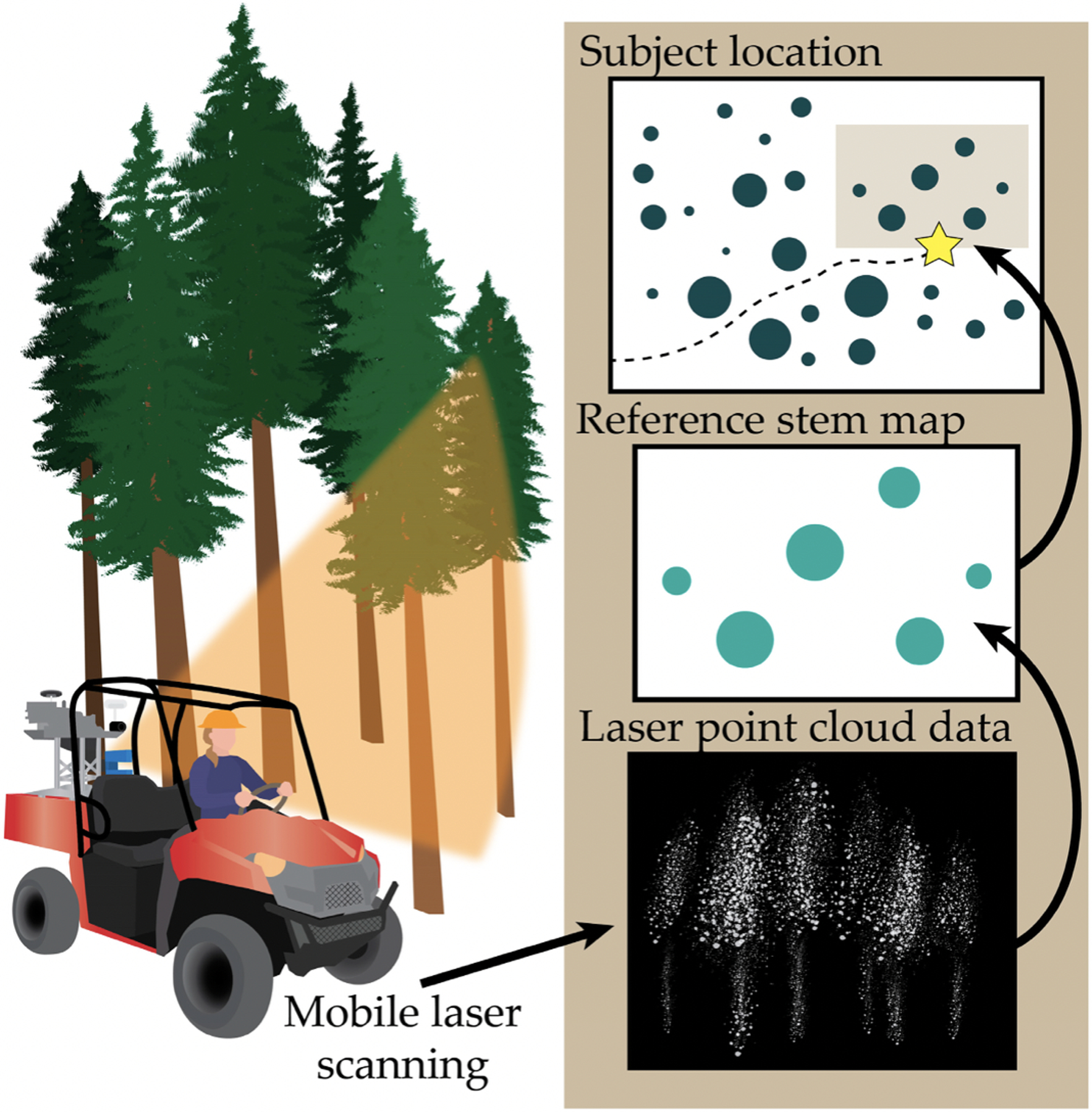
Simultaneous Location and Mapping (SLAM) techniques in the forest (based on Qian et al.) [7]. First, reference trees with known diameters must be mapped with their GNSS coordinates (not shown). Then, mobile laser scanning equipment (e.g., LiDAR) scans an area of interest in the woods (**left**). From the LiDAR point cloud data (**bottom right**), a stem distribution map is generated (middle right), which is compared to the reference tree map (**top right**) and in turn used to determine position. In practice, certainty in the map and location estimates improves with repeated passes and multiple perspectives.

SLAM involves creating a map for an unknown environment while simultaneously determining an agent’s location within it [108]. First, in landmark extraction, features of the surrounding environment are identified and mapped in relation to one another. For example, a laser scanner mounted on a mobile agent such as a robot or piece of equipment obtains a range of measurements about the surroundings to determine distances between landmarks; these landmarks are used simultaneously to localize the agent within that environment. In data association, different scans from landmark extraction are matched to increase knowledge of the mobile agent’s position. The agent’s state in the space is estimated through combined landmark observations and recorded odometry data and then continuously updated, forming a real-time map [108]. While this type of localization works best indoors, it has been deployed in forestry experimentally. For example, Hyyppä et al. used SLAM with Google Tango to extract tree diameter measurements and stem curve information for forest inventory [109], and SLAM techniques have also been paired with analysis of Light Detection and Ranging (LiDAR) data for high accuracy stem mapping (Figure 7) [7]. It is important to note that SLAM is defined as a concept rather than a single algorithm [108].

SLAM techniques are capable of sub-meter accuracy in forest environments. For example, using Google Tango, which employs color and depth cameras (RGB-D), inertial sensors, and SLAM, Tomaštík et al. demonstrated root mean square errors (RMSE) of 0.2 to 1 m under the canopy [110]. Tang et al. reported similar accuracies for SLAM combined with an inertial measurement unit (IMU) in a small-footprint LiDAR, with RMSE values as low as 0.16 m in dense forest conditions [6]. The Tang et al. study observed slightly higher errors (1.73 m) in open forests, however, which have fewer features for landmark extraction. SLAM may be integrated with GNSS/INS for even greater accuracy. Qian et al. reported horizontal positioning accuracy of 6 cm when incorporating bearing and velocity data from GNSS/INS systems, representing an 86% improvement from using GNSS/INS alone [7].

While SLAM is used widely with robots and is of interest in development of autonomous forest machines equipped with environmental sensory recognition such as LiDAR, machine vision, or acoustic sensors, the concept of *map-matching* is a related concept that may not necessarily require direct sensory recognition of the local, surrounding environment to aid navigation. Rather, in map matching, an existing map, such as navigational applications (e.g., Google Maps) are used as the basis for estimating the location or trajectory of individuals or pieces of equipment locally based on (1) known, approximate location (from a GNSS receiver, for example), and (2) the location and heading of that position relative to known map features such as roads or trails [111,112]. In that sense, previously derived forest map information such as stem maps derived from earlier airborne LiDAR acquisition, could also potentially be used for localization if that prior spatial information is present on the vehicle or machine’s onboard computer.

### 2.9. Bluetooth, BLE, and ANT

Various technologies used for wireless data exchange, such as Bluetooth, BLE, and ANT, can also serve as positioning techniques. Bluetooth, a local wireless communications standard operating at UHF wavelengths (2.4 gigahertz (GHz)), is commonly used to exchange data and connect devices (e.g., wireless headsets, keyboards, controllers, etc.) [5,26,113], but can also provide localization and proximity awareness to users. For example, Bluetooth has been successfully incorporated into hazard proximity detection and real-time alert systems to improve safety on construction sites [113]. As described in the UWB section above, positioning is based on distances from known locations of anchor devices and access points, calculated from RSSI measurements and radio propagation models [114–116]. Bluetooth is relatively inexpensive and easy to use, but high energy requirements can quickly drain the associated device’s battery [5,113]. Alternatively, BLE increases battery life by exchanging small volumes of data interspersed with periods of little to no energy consumption [26]. BLE can accurately position within approximately 2–5 m indoors [117], but operates at shorter ranges (up to 50 m) [26] compared to Bluetooth (up to 100 m for version 4.x, and up to 200 m for version 5.0) (Table 1) [118]. ANT, another local communication standard, uses deep-sleep modes to reduce power consumption but is also limited to shorter ranges (up to 30 m) [119–121].

In natural resources, Bluetooth is currently used primarily for transmitting raw data to mobile devices, such as from fitness monitors on forestry workers [122] (Figure 2, panel E), or from environmental sensors like the Kestrel^™^ weather meter [123].

### 2.10. RFID and Acoustic Positioning

RFID entails communication between an antenna and electronic tags via radio waves. Passive RFID, primarily used for tracking objects, can operate only at very close ranges of 1–3 m [5], whereas active RFID used for positioning has a much greater range of 15 m to 1 km (Table 1) [124–127]. RFID tagging is of increasing interest in precision forest management for tracking forest products through the supply chain [124]. By monitoring individual logs, companies gain a greater understanding of the quality and quantity of wood being processed from diverse sources [128]. RFID systems also help document product chain of custody to support sustainable forestry certification [129].

**Figure 8.**
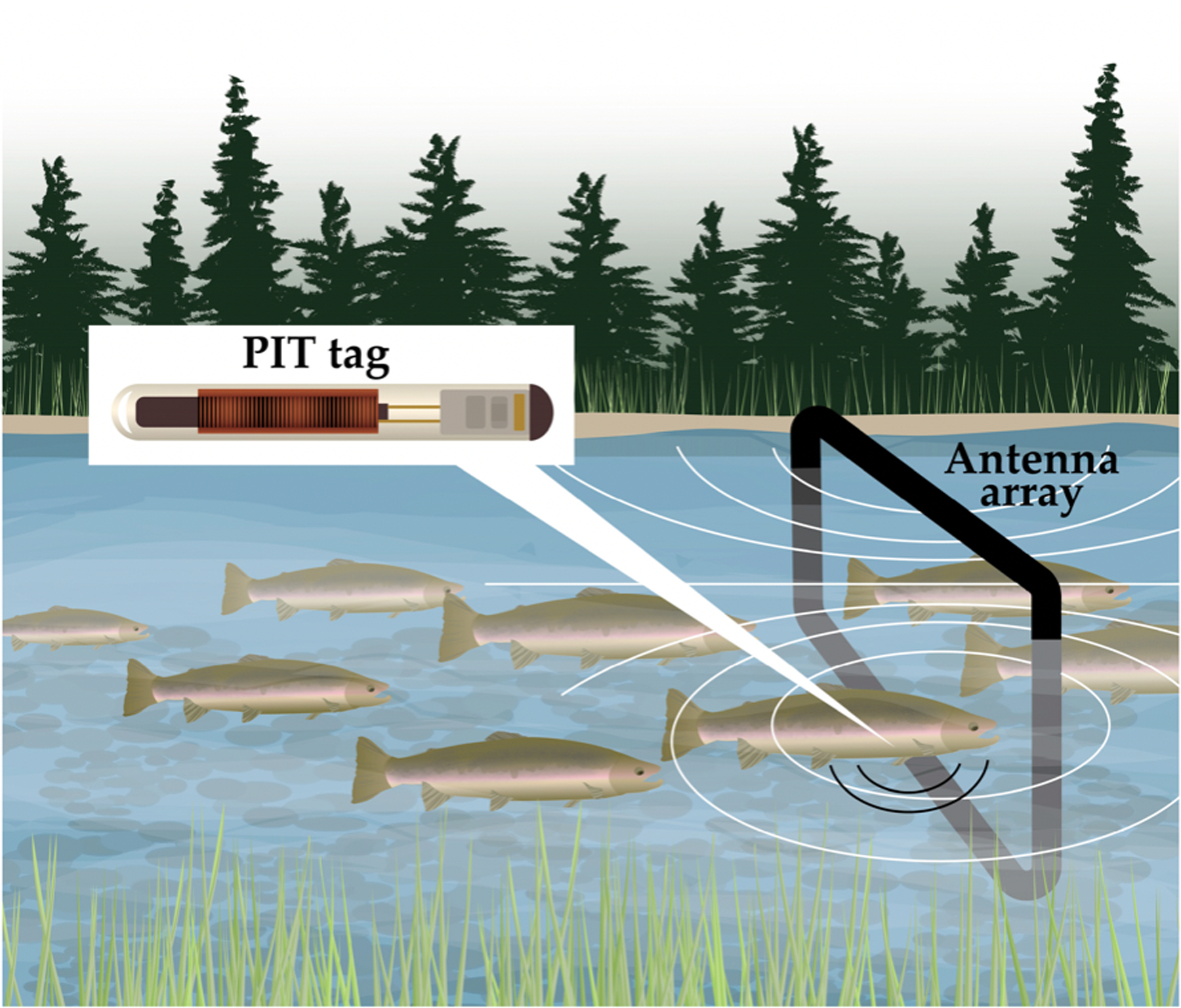
Example of passive integrated telemetry in fisheries management. The antenna array generates an electromagnetic field (white lines). A subcutaneous passive integrated transponder (PIT) tag inside the trout re-emits the array’s signal (black lines) as it passes through the field, providing information on PIT tag number, time, and direction of movement. Arrays may be staged at various locations along the stream reach to characterize, for example, the extent of fish passage.

RFID technology is an important component of biotelemetry, which has been used to study and monitor fish at multiple spatial and temporal scales for decades [96,130–133]. Trends in miniaturization and reduced prices have led to rapid increases in application, spatial extent, and number of telemetered fishes, which has resulted in big data needs for data transmission, management, and analytics. Three technologies are widely used in fish telemetry: (1) passive radio telemetry with passive integrated transponder (PIT) tags (Figure 8) [134], (2) active radio telemetry (i.e., radio tags), and (3) active acoustic telemetry with tags producing ultrasonic signals that propagate through water and are received by hydrophones [132]. PIT tags are “passive” because they do not require a battery [134–136]. Two separate PIT technologies have been applied in fish monitoring. Full-duplex (FDX) antennas produce an electromagnetic field with an antenna coil, which powers the tag directly to transmit a coded radio signal back to the PIT antenna, with simultaneous charging and listening by the antenna. Half-duplex (HDX) PIT antennas create a field that charges a capacitor in the tag, which then transmits a stronger signal back to the PIT antenna, which alternates between charge and listen modes. HDX PIT tags thus have longer read ranges but are larger. FDX systems simultaneously transmit and receive signals at up to 1800 Hz whereas HDX alternate between transmit and receive modes at up to about 840 Hz.

Biotelemetry studies have addressed questions of fish behavior, movement and migration, energetics and stress response, estimation of mortality and survival rate, habitat use, response to angling, and monitoring of invasive species (see recent reviews in Adams et al. [132], Cooke et al. [135], Hussey et al. [96], and Crossin et al. [133]). Aquatic studies are not limited to economically important fishes (e.g., Breen et al. [137]) and have also included larger aquatic invertebrates (e.g., crayfish [138] and freshwater mussels [139,140]). Integration of depth sensors, temperature sensors, and accelerometers to telemetry tags promises a new wave of detailed studies [96]. For instance, Fuchs et al. used a prototype commercial radio tag with accelerometers to develop protocols to quantify spawning behavior in steelhead trout in turbid rivers during snow-melt runoff [141].

Radio-telemetry tags use an onboard battery to actively transmit identity, and in some tags, data on depth, temperature, acceleration, or other parameters. Range is typically greater than 1000 m when tags are near the surface of freshwaters, but effectively zero below 10 m [135]. Commercially available acoustic tags also use battery power to actively transmit identity and may also transmit other parameters such as depth. These can be used effectively in deep habitats such as lakes and slow rivers but are limited in rapidly flowing waters (i.e., those with entrained air). Monitoring is primarily accomplished by either fixed sites or mobile tracking for PIT tags and both types of active tags [132,135]. Fixed sites for radio receivers may be on alternating current (AC) hard power, solar, or battery. Received data can be manually downloaded or transmitted via cellular or satellite networks. Hydrophones can be deployed in a similar manner, or using autonomous nodes deployed on the bottom of waterbodies with up-looking hydrophones. Nodes are recovered using a remote-controlled release and are manually downloaded weekly to monthly. PIT antennas are relatively power intensive because the antenna both transmits and receives; larger antennas are frequently on hard AC power and have access to internet uplinks for data transmission. Remote PIT sites are usually powered by solar or propane generators, with manual downloading or data transmission via cellular or satellite uplink (e.g., Achord et al. [142]). Mobile tracking for all three tag technologies can be via hand-held or vehicle-mounted unit, some with integrated GPS.

Biotelemetry studies generate large data sets, with hundreds to thousands of fish tagged, 100s to 1,000,000s of records per individual fish, and data from 10 to over 100 individual fixed receiver sites. Substantial data processing prior to the interpretation of movement records is required, such as critical quality assurance/quality control (QA/QC) steps including the estimation of detection efficiency for each receiver [132] and protocols for eliminating “noise” records. Monitoring can include project-specific receiver arrays or, increasingly, multi-investigator, multi-agency monitoring networks [96] such as the Columbia Basin PIT Tag Information System (PTAGIS; ptagis.org). PTAGIS operates large scale PIT-detection sites, develops software to collect metadata on fishes tagged by regional organizations, and provides a publicly accessible database of PIT tag detections on PTAGIS and other PIT detection sites across a watershed approximately the size of France via a website, which reports detections in real-time. In 2017 alone, 36 organizations contributed records to PTAGIS for over 2 million PIT tagged fish (and a cumulative total of over 45 million tagged since 1987) and recorded 12.2 million detections at 309 antenna sites for 822,000 individual fish [143]. On the data management side, over 422,000 data files were processed in 2017, and nearly 500 researchers/users executed 342,000 database queries returning over 7 billion rows of data [143].

### 2.11. Barcodes and QR Codes

Asset and inventory tracking tools such as one-dimensional barcodes, two-dimensional barcodes (QR codes), and Near Field Communication (NFC) tags, that are used ubiquitously for local and global commerce also provide a method of positioning and navigation in forests. QR codes are similar to barcodes but store information in two dimensions in order to expand data storage capacity [4]. NFC tags are based on RFID technology and offer two-directional short-range wireless communication [144]. Users can interact with these technologies via the sensors or camera in mobile devices, both to record and retrieve information [144–146], such as local maps or nearby landmarks [147–149]. Activated codes or tags can also be used to establish a known time and location of access (e.g., Google Analytics) and thereby inform map-matching of resources across multiple waypoints.

**Figure 9.**
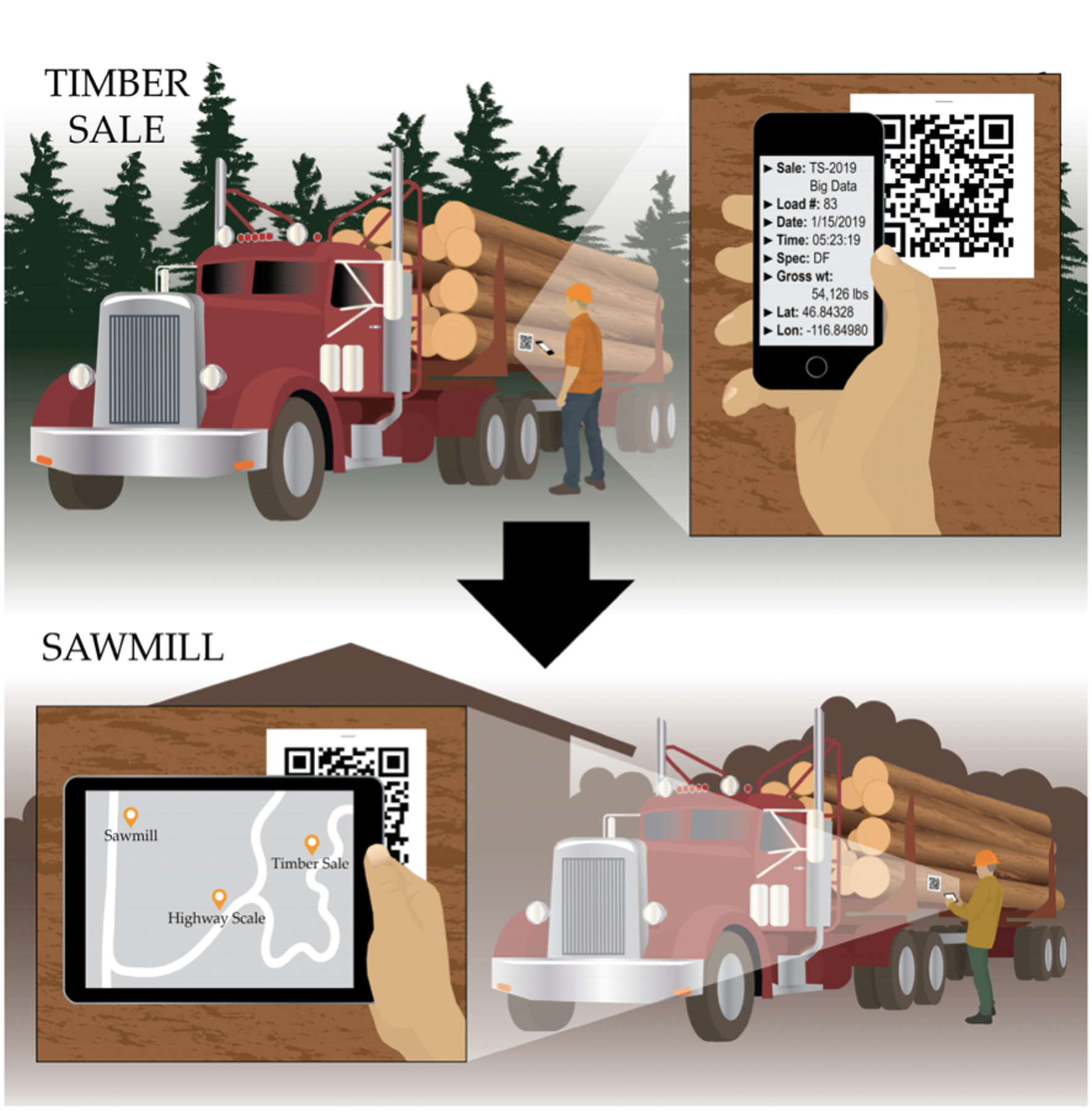
Quick response (QR) codes printed at the jobsite with log load information are scanned again at multiple waypoints, such as truck weight scales on a state highway and the sawmill. Chain of custody information, like the weight of the load, can be monitored, and, insofar as the time and place of the truck is recorded on a map multiple times, this method serves as a form of positioning.

Barcode tracking technologies such as QR codes are used in forestry to monitor log load information from stump to mill (Figure 9) [3,4]. QR codes are applied to log ends printed on paper [3] or marked directly on the wood with lasers or paint [4,150]. With higher storage capacities than simple, one-dimensional barcodes [4], QR codes can store load details such as harvest unit location, species and product sorts, bill of sale documents, or environmental certification status [3,150]. Information can be accessed with a mobile device like a smartphone [150]. QR codes are cost-effective, accurate, weather-resistant, and will not hinder downstream processing [3,150]. In addition to storing data, they can trace where products have been throughout each phase of the supply chain [4]. When used in conjunction with cloud-based data management systems, QR codes can be used to track logs in real-time [3,150]. Thus, QR codes can help monitor the movement of resources to improve efficiency and logistical planning, as well as to document chain of custody [3,4,150].

QR codes have also been used for wildland fire applications to disperse information in communities faced with fire. For example, QR codes printed on business cards or flyers can connect the public to online resources with fire status updates, such as social media websites and webcams [151].

### 2.12. Video Object Detection and Relative Positioning Methods

Similar to the map-matching methods described earlier in the context of SLAM, object detection using video imagery can also be thought of as a positioning method for moving objects or other resources. The sequential video frames for a known landscape field of view provide a reference map, and manual (visual) observation or automated detection of people, wildlife, or other phenomena within that scene can be used to estimate location. A variety of algorithms and techniques exist for identifying and delineating the background scene from one or more foreground objects based on shapes, color, contrast, and other image features [152–155]. In forest management, video object detection methods have received the majority of attention in the context of detecting wildland fire [156–158] and smoke [159–161]. As unmanned aerial vehicles (UAVs) are used more widely in fire management, fire and smoke detection from UAV-mounted video is becoming more prevalent (e.g., Yuan et al. [162]). Positioning of logging workers based on field safety vest characteristics detected by cameras mounted on the skyline carriage was proposed by Keefe et al. to increase situational awareness for equipment operators [163].

**Figure 10.**
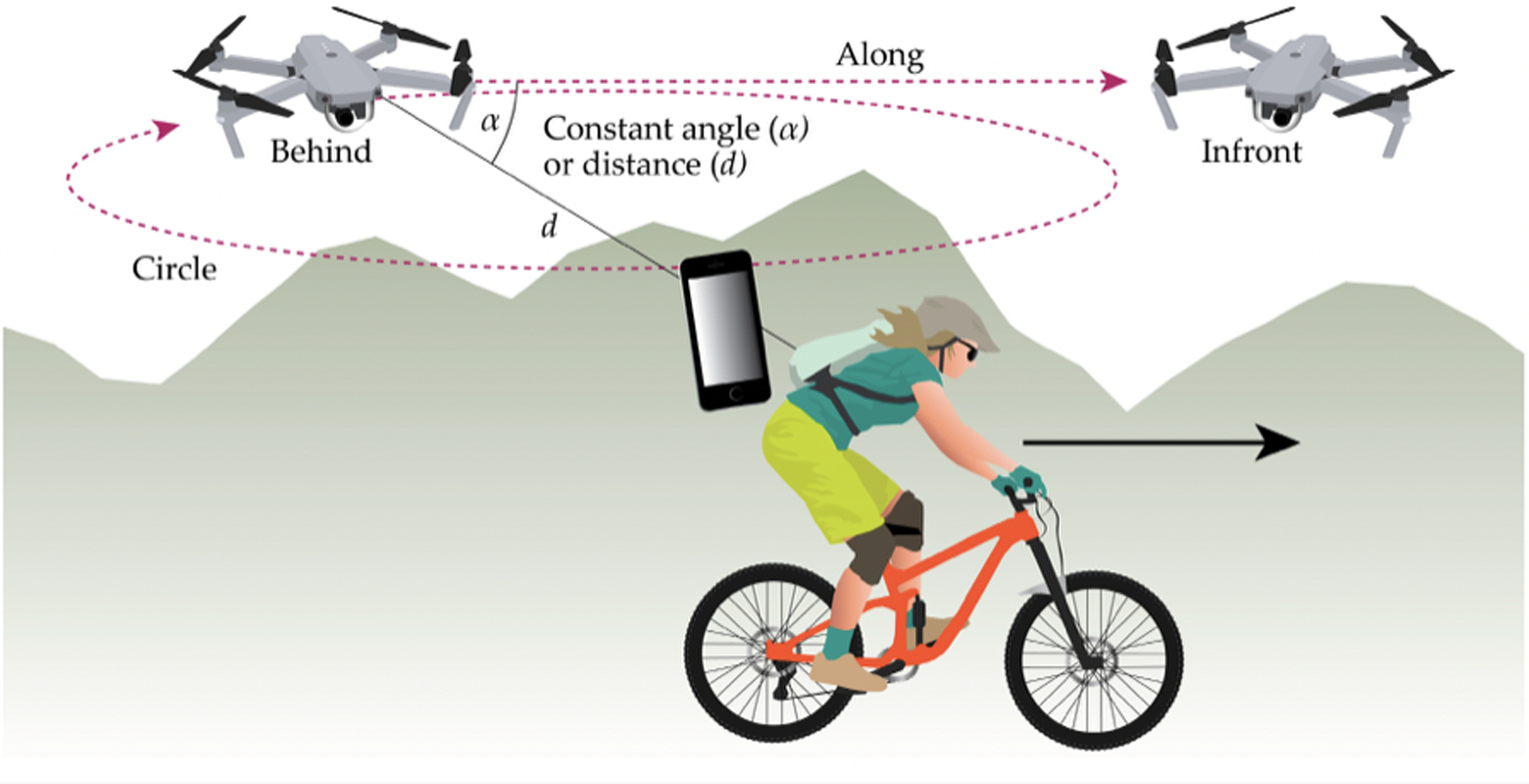
Relative positioning for a quadcopter unmanned aerial vehicle (UAV) with following capabilities. The device’s onboard computer navigation can be programmed by the user to maintain a fixed distance and orientation relative to a moving object, person, or animal. Infrared sensors, machine vision with pattern recognition, acoustic positioning or other methods may be used to infer the UAV position relative to the subject. In this example, the drone positions itself relative to the smartphone’s GNSS coordinates.

In addition to the various first-order positioning methods described thus far, it is important to note the existence and growing importance of technologies that use either sensors, video imagery, or other methods to position themselves *relative* to other objects, whether stationary or in motion. This concept is important in automation and also in recreation applications. Co-location relative to other mobile devices is distinct from independently positioning multiple phones or other mobile devices [164]. A simple example of relative positioning is the ability of follower drones to maintain a moving position at a constant distance and orientation relative to a person, animal, or piece of equipment being monitored (Figure 10) [165,166]. Visual relative localization is also used to configure swarms of UAVs flying in formation or in other coordinated activities [167]. Acoustic signaling is another form of relative localization that has also been used to collocate UAV swarms based on follower positions relative to a leader or peer [168]. This approach may also have applications in recreational use of UAVs and equipment automation in forests.

## 3. Accuracy and Range of Available PNT Technologies

As discussed in the previous sections, the effective ranges and accuracies of different devices vary widely. Table 1 summarizes the approximate communication ranges of technologies discussed in this paper with corresponding references. It is important to note that several of the technologies or protocols discussed, such as Bluetooth or UHF/VHF, are both communication frequencies and methods of estimating position. As described in previous sections, position estimation using these approaches may involve initial development of a regression model to predict distance to a signal transmitter as a function of path loss (or RSSI), or other methods such as TOA and TDOA described for UWB positioning. For this reason, the effective transmission range is closely related to positioning capabilities. In some cases, frequencies or communication protocols may be used for (1) communication of data; (2) position estimation based on localization; and (3) both of the preceding applications. Additionally, although we have discussed two- and three-dimensional positioning extensively, one-dimensional (1D) proximity (distance from a point in any direction) is also commonly deployed for Bluetooth and RFID devices (e.g., phone apps and small Bluetooth devices for locating keys or tagging pieces of equipment).

Accuracies reported in Table 1 are for static positions rather than objects, people, or equipment in motion, with the exception of GNSS-RF (as indicated). As research on PNT applications in forestry, wildland fire, and additional natural resource management concentrations emerge, it is important that researchers and practitioners develop new analytical methods, or adopt methods used in other fields, to better quantify device error and accuracy in ways that integrate both location and velocity. This is particularly important as software applications are increasingly built around equipment and human resources in motion.

## 4. Location-Based Services, the Internet of Things, Wearable Technology, and Big Data

Having reviewed a wide range of positioning approaches that may be useful for analytics in forest and fire management, we now consider and review broader concepts from other fields including, among others, data science, business marketing, and computer science. Our objective in doing so is to (1) provide an overview and review of these emerging concepts; and (2) consider potential applications of their deployment in forest science and management. We are particularly interested in the potential for these somewhat abstract concepts, when integrated with the reality of emerging, high-accuracy positioning systems discussed previously, to foster new advancements in analytics that support production efficiency, health and safety, and automation in natural resources.

### 4.1. Location-Based Services

**Figure 11.**
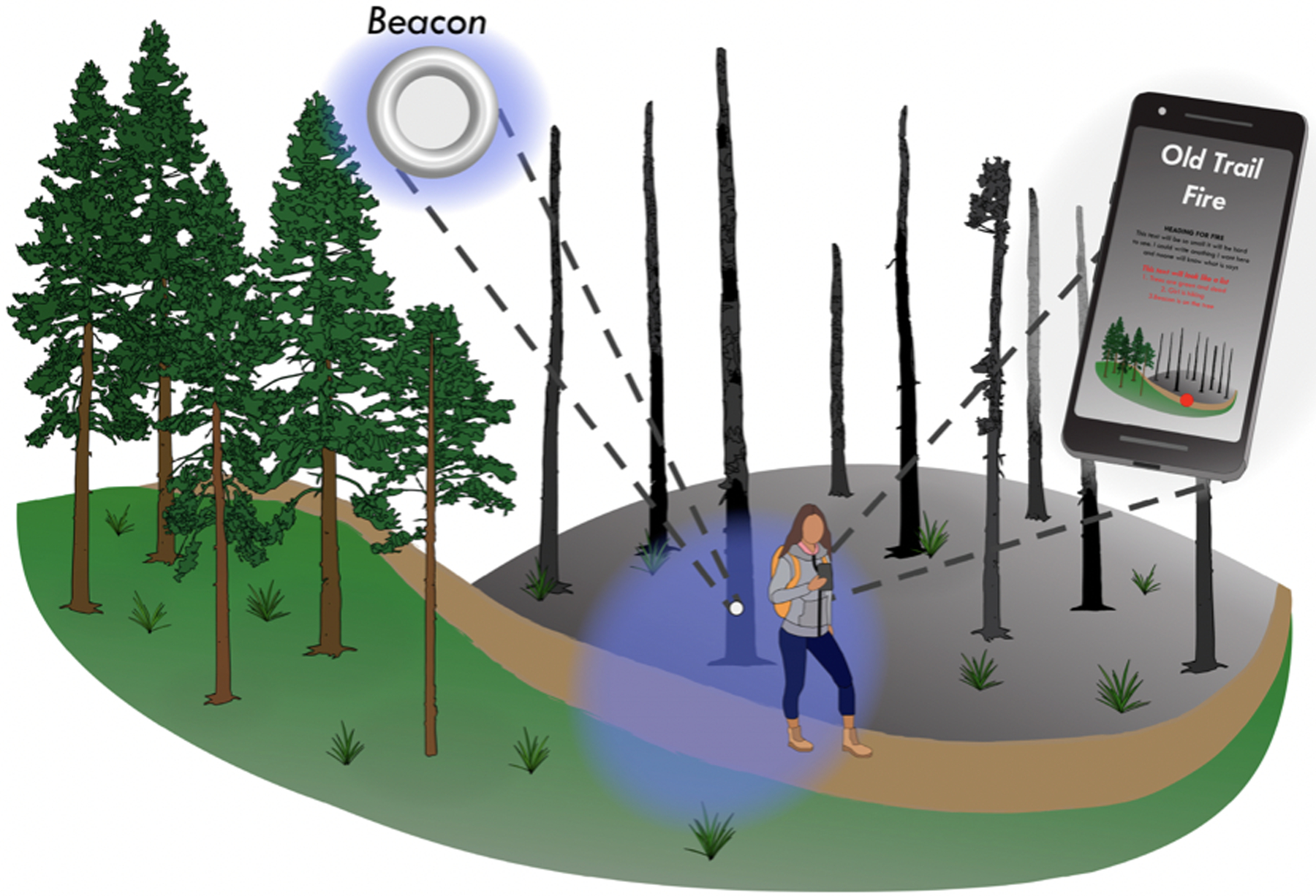
In location-based service applications for mobile devices, proximity to Bluetooth beacon signals trigger the sharing of information provided in smartphone applications. Mobile applications may also then develop metrics based on how many users have passed a beacon location, or modify information provided based on a sequence of actions taken by users as they interact with multiple beacons. LBS can also base information on geographic coordinates (GNSS) or proximity to map-based features.

Location-based services (LBS) refer to services that account for the real-time, geographic positions of a person or mobile object, as determined by RF, Bluetooth or BLE, GNSS, assisted GNSS (perpetual locating of a device), or broadband satellite networks [169]. They may include reactive services activated by a user, such as mapping a location or displaying nearby points of interest, or proactive services automatically initiated when a user enters a specified area [170]. LBS require a real-time exchange of data between a user and a service provider over a wireless network, typically through a mobile device like a smartphone [169–171]. Data may be used for location-tracking of others, such as finding a friend or location-aware services based on apps making use of the device’s position to trigger some action or notification. A simple example of LBS is the updating of date and time on a mobile device associated with the user crossing into a time zone [172,173]. With the availability of consumer-grade beacon transmitters such as Apple’s iBeacon^™^ and Google Eddystone^™^ beacons, any object can transmit location-based information to a mobile device using BLE [174]. This approach, though developed for retail marketing in urban areas, can also be integrated into forest recreation, education and outreach, and logistics applications using outdoor beacons with currently available software development platforms (Figure 11).

LBS have applications in navigation (e.g., Google Maps), fleet and asset management, personal safety, mobile marketing, mobile gaming, points of interest (e.g., finding nearby stores or restaurants), and enhanced emergency services (e.g., enabling the tracing of 911 calls from mobile phones by polling the devices’ GNSS coordinates) [169,170,173,175]. De Longueville et al. proposed using Twitter messages as a form of LBS to gather spatial and temporal information about fire movement in real-time [176].

A recurring consideration for LBS research and applications is the issue of privacy [169,170,172,173,175,177] due to the potential for surmising a variety of personal information from a user’s location. For instance, user queries may reveal sensitive information on health, lifestyle preferences, or political and religious affiliations [175]. Privacy may be protected through data encryption [169,175] or location anonymity techniques, which send a user’s location along with false “dummy” locations, so service providers do not know the user’s true position [177].

### 4.2. Geofences

Geofences, virtual boundaries, virtual fences, and proximity alerts are names for closely related concepts in which an action or alert is triggered when an object with a mobile device or other transponder crosses a geographic boundary associated with a pre-defined area or point of interest (POI) [178,179]. Geofence boundaries may be defined as a radius around a POI or by an irregular polygon, typically based on GNSS coordinates [178]. Geofencing has been suggested for a variety of spatial-awareness applications, including location-based services for tourism (e.g., recommending nearby sights) and advertising (e.g., offering coupons to passers-by) [179], fleet monitoring and management (e.g., tracking shipments) [178], smart cooling (e.g., activating fans only when individuals are present) [180], and emergency response (e.g., keeping traffic lights green when an ambulance or police vehicle approaches an intersection) [181].

In natural resources, geofences may help delineate hazardous areas. For example, Sheppard et al. used geofences and biotelemetry devices to alert wind farm operators of the proximity of large birds to reduce collisions [182]. Geofences have also been evaluated for forestry applications to mark unsafe working areas around equipment hazards or snags (dead trees) on active logging operations [20,21,85]. Zimbelman et al. assessed errors associated with mobile geofences that move with a dynamic, point hazard, such as a hand faller [22]. In this case, the geofence represents a hazardous area for others, but can also define safe zones for the hand faller, triggering an alert when he or she approaches another geofence, such as around a second hand faller.

**Figure 12.**
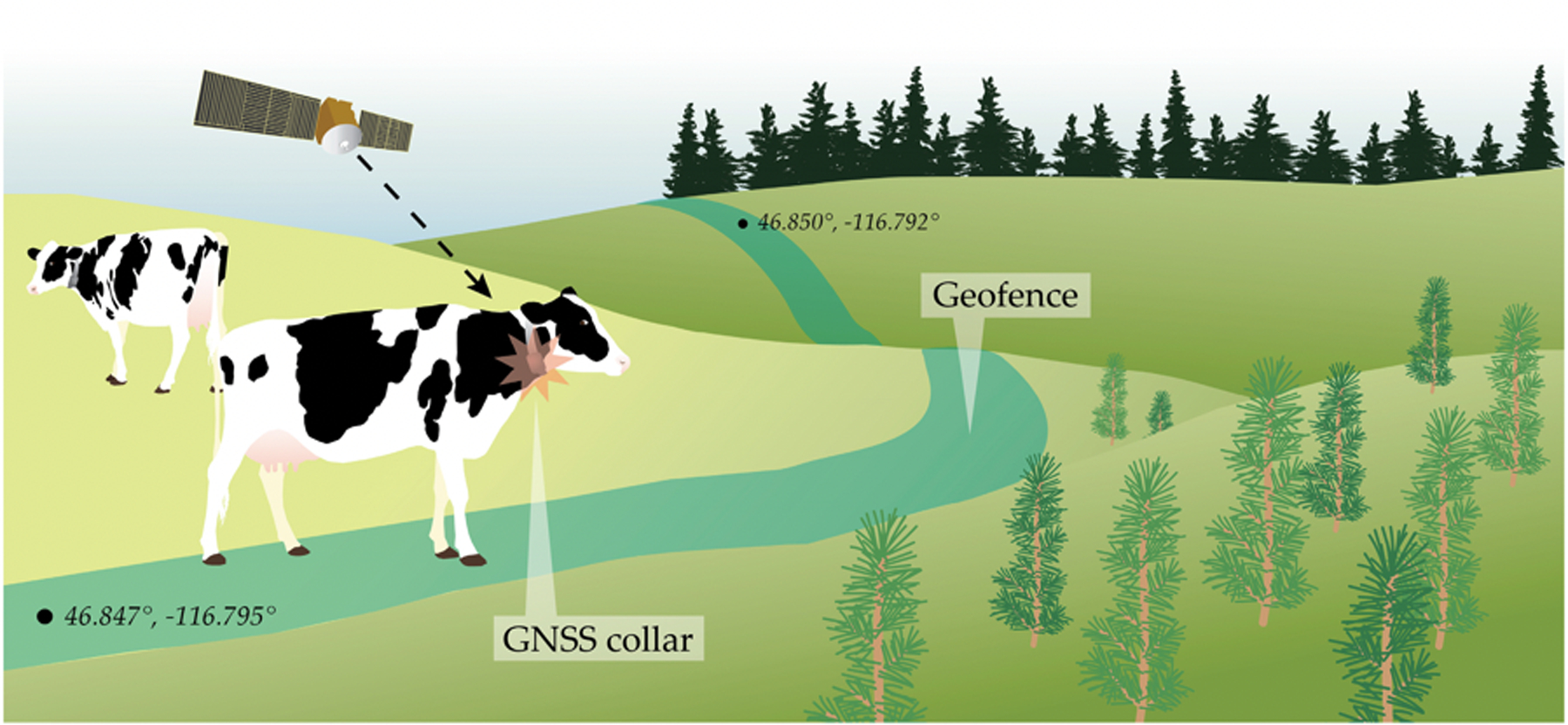
Example use of a geofence to separate grazing from newly planted conifer seedlings. The virtual fence is defined by a series of GNSS coordinates. When a GNSS collar worn by livestock approaches the boundary, an alerting sound or sensation is triggered.

Virtual fences are also used as animal containment systems and have been deployed widely in rangeland science to manage animal movement [183–185]. An alternative to traditional, static fencing, virtual boundaries can promote a variety of management goals, such as monitoring animal movement [186], separating individuals [185], limiting herbivory [184], protecting riparian habitats [185], reducing soil degradation [183], and relocating herds [183,185]. Although virtual fencing is not infallible [187,188] and may not be appropriate for non-social species [186], it has several advantages over traditional fencing, including the capacity to change spatially and temporally [184–186] and the ability to cover remote or difficult terrain [185]. Instead of trigging an alert, crossing a boundary administers a sensory stimulus, typically through a collar, aimed at altering the animal’s behavior (i.e., further movement past the fence) [183,184] (Figure 12). The stimulus may be a sound, vibration, light pulse, shock, or combination of cues that cause irritation or annoyance to train an animal to avoid an area [183,185,187].

### 4.3. Wearable Technology

Wearable technology is a general term that refers to miniaturized sensors such as accelerometers, gyroscopes, and magnetometers built into watches, clothing, or other accessories that can be worn comfortably on the body to enable real-time, unobtrusive monitoring of physical metrics [189–191]. The first wearable technology that received significant media attention was the Wearable Motherboard, a smart shirt designed to monitor body vital signs in combat situations [189]. Wearable technology has since been deployed in a variety of health and fitness applications, predominately related to the continual, long-term observation of patient health status remotely [190]. Individuals with chronic conditions such as Parkinson’s disease can wear unobtrusive sensors in the home so that health care providers can more accurately assess physical or physiological changes and recommend treatment or medication [190]. Wearable technology can also facilitate disease diagnosis and preventative care [189,190]. Since physiological data can be collected and processed in real-time in networked environments, wearable technology can initiate medical response in the case of sudden emergencies like cardiac arrest or epileptic seizures.

Wearable technology can also refer more generally to any computerized device worn on the body. For instance, eye-wearable devices such as Google Glass^™^ deliver visual information to a user via a small screen. Unlike many other wearable device applications that focus on collecting data, eye-wearable technology is typically employed as a conduit for conveying data to a user, such as hands-free instruction in medical training or vehicle maintenance [191,192].

Bowen et al. monitored logger work activities and sleep patterns with fitness and sleep bands in order to evaluate worker health and identify hazardous work conditions [193]. The Bowen et al. study highlighted several considerations for deploying wearable sensors in forestry applications, including long battery life, little to no operation requirements by users, and low-cost due to the potential for devices being dislodged and lost during work activities [193]. In another study of loggers, Bowen et al. collected heart rate and step count data using Fitbits^™^ in conjunction with in-situ reaction time measurements in an attempt to predict worker fatigue [194]. The 2017 study illustrates the variability of physiological data among individuals and the importance of context for understanding and interpreting field data. Additionally, due to the personal nature of information collected with wearable technology, privacy and the ramifications of sharing such data should be carefully considered [194].

### 4.4. Activity Recognition

The concept of activity recognition refers to use of the wearable devices and sensors described in the previous section to quantify and monitor human activity, with applications ranging from fitness and health to military and security [16,17]. Activity can be surmised from interactions between users and sensor-enabled objects such as in smart houses, or can be predicted based on measurements collected by wearable sensors [16]. Wearable sensors can detect user movement (e.g., walking, running, standing), location, or physiological traits (e.g., heart rate, respiration), as well as provide context about a user’s environment (e.g., temperature, humidity, audio level, light level) [16,18]. They facilitate continuous, real-time activity monitoring, but are easily removable when users do not want their activities recorded [17]. Smartphones are a popular choice for wearable, sensor-based activity recognition [11–13]. They are relatively unobtrusive and widely used, and they include several internal sensors. Keefe et al. developed activity recognition models to characterize the work activities of professional timber fallers based only on smartphone sensor data [14]. Models correctly characterized real-time activities of the loggers working on industrial cable logging operations such as felling trees with chainsaws, clearing brush, and walking between trees [14].

Smartphones typically include GNSS chips, triaxial accelerometers which record movement in three directions, gyroscopes to determine orientation, microphones that record sound or measure sound levels, cameras, light sensors, digital thermometers, and magnetometers to measure compass direction [16,18]. Because they also have internal microcomputers, smartphones can process sensor data directly using existing or custom apps. This reduces the need to transmit primary data through a wireless network, which can slow down real-time applications considerably [16]. Smartwatches and other wrist-based sensors are also now used extensively in recreation, sports and exercise [8–10,195]. An important consideration for employing smartphones and other wireless sensors to collect continuous sensor data, however, is device battery life, especially when multiple or energy-draining (e.g., GNSS) sensors are engaged [16,17]. In the future, activity recognition may be achieved using different methodologies without sensor-based devices, however. For instance, Wang et al. demonstrated how disruptions in WiFi signals could be used to model human movement speeds, which in turn could predict activity with 96% accuracy [196].

**Figure 13.**
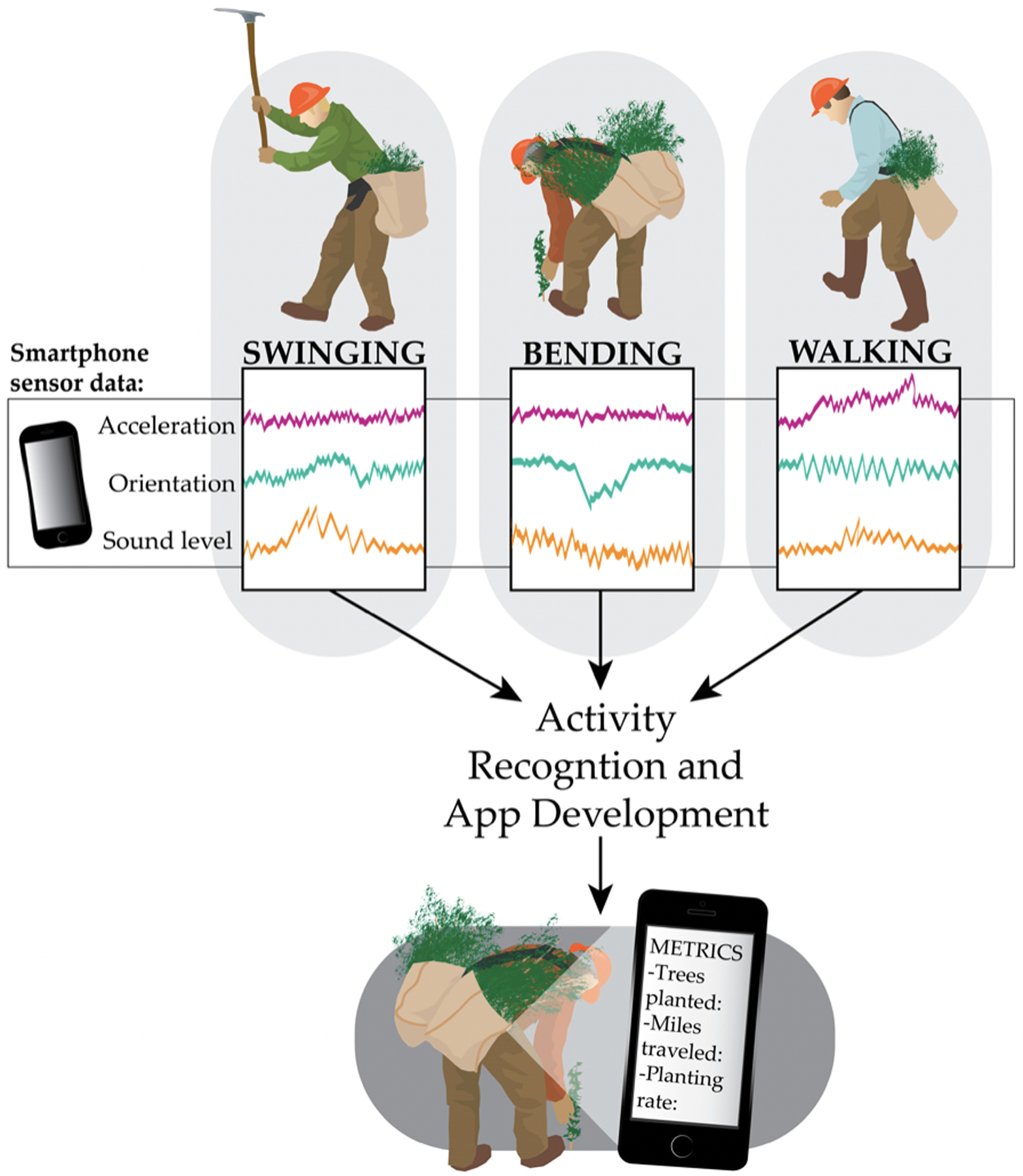
Activity recognition modeling and application, using tree planting as an example. Initial sensor data is collected during completion of specified activities: swinging a hoedad planting tool, bending to place a seedling, or walking to the next site. Patterns in the raw sensor data are used to compute activity recognition, which can then be incorporated into apps for use on mobile devices in the field to quantify work activities, such as number of trees planted. This figure is adapted from Keefe et al. [14].

To predict activities, initial sensor measurements associated with a particular activity must be collected and then processed with feature extraction, which involves filtering out relevant information from the raw sensor data (Figure 13) [16,17]. Both time and frequency domain features, such as mean, standard deviation, variance, and Fast Fourier Transform and Discrete Cosine Transform coefficients, may be extracted with a moving window approach [13,14,197]. Using a moving window, subsets of the data are defined by the window size (i.e., length of time) and this window is advanced in pre-determined increments. In this way, features are calculated using a new subset of the data defined each time the window advances [14]. A machine learning model is generated based on patterns in the extracted features and used to infer activity based on sensor measurements.

Various machine learning algorithms can be used to develop predictive activity recognition models, such as Decision Trees, Support Vector Machines, K Nearest Neighbors, and Naïve Bayes [13,197]. Device orientation and location on the body are also important considerations that affect the accuracy of predictive models [13,197–199]. Ideally, activity models are individual-specific (subject dependent), to account for nuances in movement among users, but for practical reasons, may generalize an activity across a group of users (subject independent) [9,16]. Activity recognition models may then be implemented on a mobile device to provide near real-time classification. As smartphones have become increasingly powerful, the concept of “online” activity recognition has become an important research area, referring to implementing the entire classification process (i.e., data collection, pre-processing, feature extraction, and classification) locally on the device [13,197].

### 4.5. Mesh Networking

Devices can form local networks that connect indirectly to Internet services through wireless mesh networks (WMN). In a mesh network, some devices, referred to as routers, form a wireless backbone to transmit data from users to wired access points [200]. Meshing may be full, in which all devices (nodes) are connected to every other device in the network, or partial, in which some devices connect to all and some only transmit to a single node [201]. Any device that joins the network creates additional opportunities for hops, or relay points for sending data through the network, thus increasing network coverage [200,202]. Multihopping also increases bandwidth, which is higher at shorter ranges [200,203]. Since the nodes create multiple pathways of communication, WMNs are relatively immune to single point failure, in which failure of one node disrupts communication through the whole system [200,202]. WMNs are intended for permanent or semi-permanent network connections with some wired infrastructure. In contrast, mobile ad hoc networks (MANETs), do not require any infrastructure and are intended for temporary connections of highly mobile devices with variable arrangements (topology) within the network (Figure 2, panels D and F) [200,202]. MANETs have primarily been used for military applications or specialized civilian operations, such as disaster recovery [200]; whereas, lower-cost WMNs have been applied more widely. WMNs have been used, for instance, to provide cost-effective Internet connectivity, especially for remote or scarcely populated communities [200,203], to create intelligent transportation systems which provide real-time travel information about public transit [200], to enhance communications for public safety and first response [201,203], and to improve utility company services, such as more accurate automated meter reading systems and quicker detection of gas or water leaks [203].

**Figure 14.**
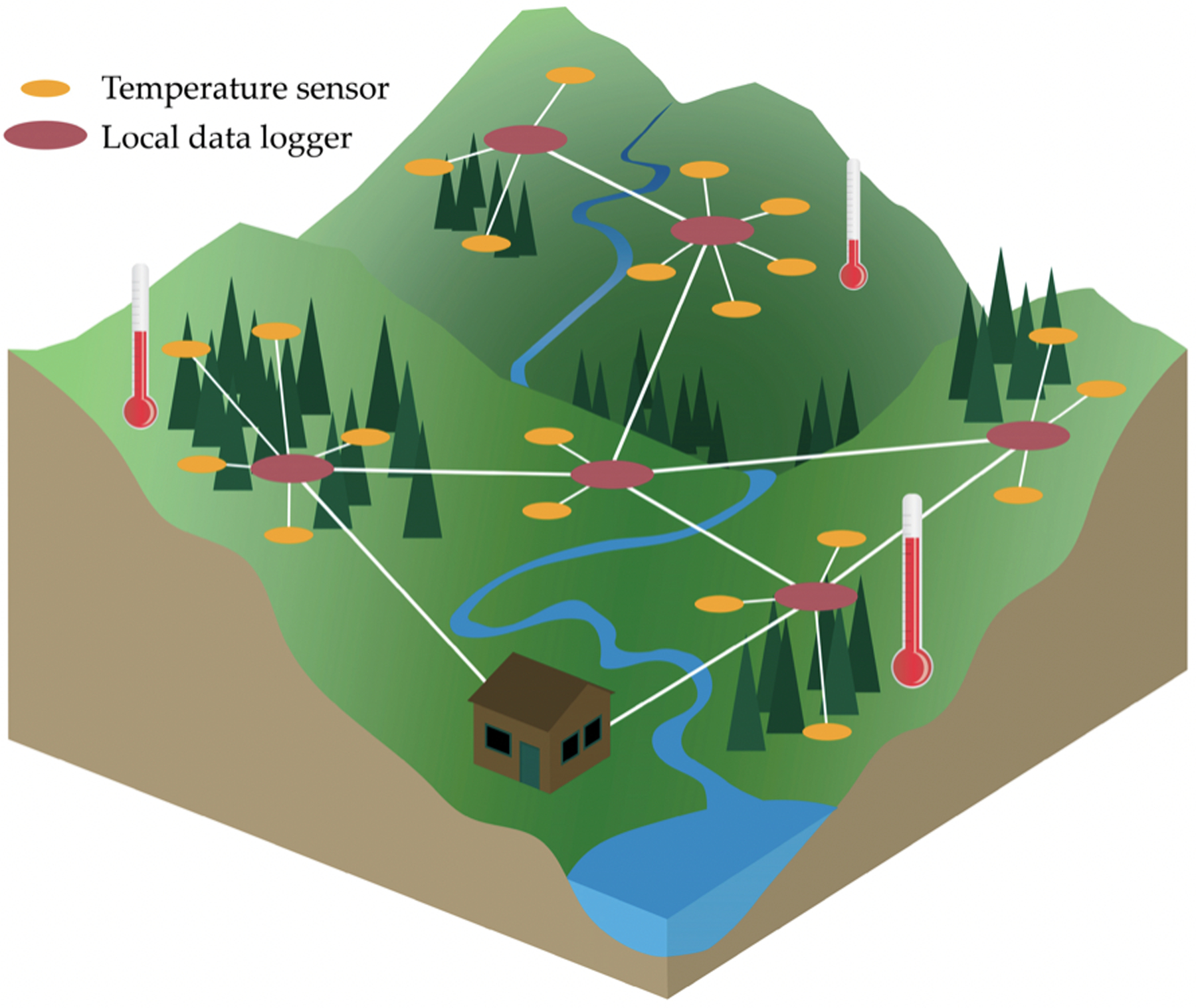
Wireless sensors in a mesh network in which individual units (the temperature sensors) transmit data to local nodes (the data loggers), which in turn transfer data to a station that is connected to outside resources via the Internet. Whereas positioning devices discussed in the early sections of this paper primarily detect the movements of people, equipment, or other resources, wireless sensor networks (WSNs) are used broadly in forest research and in environmental fields to monitor air masses, soil moisture, and other environmental factors spatially.

Wireless sensors can use mesh networking to transmit field measurements in remote areas to local data loggers, and then on to wired access points (Figure 14). In forest science, wireless sensor networks (WSNs) are used to accomplish a variety of management and safety goals, such as measuring forest health or detecting wildfires. For example, sensors collecting data on temperature, humidity, soil moisture, light intensity, nitrogen concentration, carbon dioxide levels, and canopy closure can provide remote observations of forest health [204–206]. Real-time data from environmental sensors at known GNSS coordinates may help detect and locate wildland fires, either by reporting weather conditions conducive to fire ignition and spread (e.g., temperature, humidity, wind speed, and wind direction) or by alerting a central server of the presence of smoke or infrared radiation [207–212]. Wireless sensor networks in forested environments are prone to transmission error, as radio signals are disrupted by vegetation and trees [23,209], so diagnostic tools that can identify network faults are valuable for recognizing incomplete data sets [204].

### 4.6. The Internet of Things (IoT)

The Internet of Things (IoT) encompasses a global network of interconnected physical objects that can interact with one another and with their environments to accomplish common goals [213–215]. Each object possesses a unique identifying Internet Protocol (IP) address that is recognizable via an internal computer board, RFID tag, barcode, or any other networked means of identification [213–216] Sensors associated with the object collect information about its physical environment, such as temperature, motion, or location [213,214]. Sensor data, along with objects’ identifying addresses, are then transferred through wireless networks to a database for storage and processing. This big data is typically too immense for physical hardware to accommodate and is thus usually transmitted to a virtual, shared cloud network, where it can be stored and processed by middleware. After processing, the middleware prepares visualization of the data to be easily interpreted and understood by end-users [213,215] for a variety of analytics.

Similar to location-based services, the IoT entails sharing potentially personal information through a wireless network. Maintaining user security and privacy, such as through data confidentiality, in which only authorized entities can access information, is a critical challenge for the IoT [213,214,217]. Another challenge is the need for enough addressing space to accommodate the billions of interconnected objects in the network, each with its own distinctive identifying address [213,214]. Additionally, the IoT requires increased processing capabilities to accommodate the immense number of service requests. Recent studies have highlighted the use of fog computing to complement cloud centers [213,218,219]. Fog devices are smaller, cheaper, and generally have fewer performance delays than cloud centers, making them the ideal choice for handling real-time services requiring immediate processing [213,219]. Fog devices can route other requests requiring extensive analysis or more permanent storage to a cloud computing center [219].

IoT technology has been applied across multiple industries. In transportation and logistics, it has been used for collision avoidance systems, augmented maps, self-driving vehicles, traffic incident information, route optimization, and delivery time status [213,214]. IoT enables industrial automation, in which computerized robots complete manufacturing tasks, from parts production to quality control. Also, with IoT, health care providers can receive real-time physiological data on patients. IoT is integral to smart home and building technology, allowing users to control appliances, electronics, lighting, security, and temperature remotely or to program activity based on conditions like time of day for lighting or current use for electrical equipment [213,214].

### 4.7. Big Data

At its simplest, big data refers to the unprecedented volume of data currently being generated as the total quantity of devices and their ability to measure and store data continues to increase over time. Since size is relative, the term also embodies the technological limitations for storing and processing digital information efficiently [220]. Based on existing, available storage capacities, big data is usually measured in terabytes, petabytes (1,024 terabytes), or even exabytes (one million terabytes) [221,222]. One terabyte is equivalent to 1500 compact discs (CDs) or 16 million Facebook photographs [221]. Definitions of big data typically also account for its high variety and high velocity [220–222]. Variety refers to the heterogenous nature of the data, which can be tabular (e.g., spreadsheets), structured, or unstructured (e.g., text, image, audio, video). Velocity denotes both the rate at which data is generated and the speed at which it needs to be analyzed to inform actions or provide services.

Diverse fields generate big data, including health (genomics, medicine, pharmaceuticals), science (astrophysics, quantum mechanics, modeling, simulation), government (statistics, campaigning), higher education (admissions, student evaluation), and business (inventory, advertising, decision-making) [223,224]. Daily interactions with objects like smartphones and other wearable technology or interactions with other people through social media sites also produce big data [221,222]. In natural resources, big data from global satellite imagery or terrestrial monitoring systems (e.g., LiDAR) can help assess environmental changes and inform management decisions [224]. Emerging individual, tree-based forest inventory, particularly using airborne, terrestrial and mobile laser scanning, to support precision forestry is expected to generate considerably larger volumes of data than conventional forest sampling [225,226].

Multiple challenges stem from the creation of big data. Technical solutions are required to meet the need for advanced data management (acquisition, storage, and transfer) and analytics (extracting useful information from the data) [220–223]. Some data sets include personal, sensitive information, such as electronic health records, which necessitate increased privacy and security measures [220,223,224]. Additionally, issues of ownership and legal rights arise over big data, such as individual information collected through social media sites or intellectual property related to pharmaceutical research [220,223,224]. Ownership also entails determining who is accountable for inaccuracies, particularly when those inaccuracies have negatives outcomes [223].

## 5. A Hierarchical Model for Processing and Sharing Data

**Figure 15.**
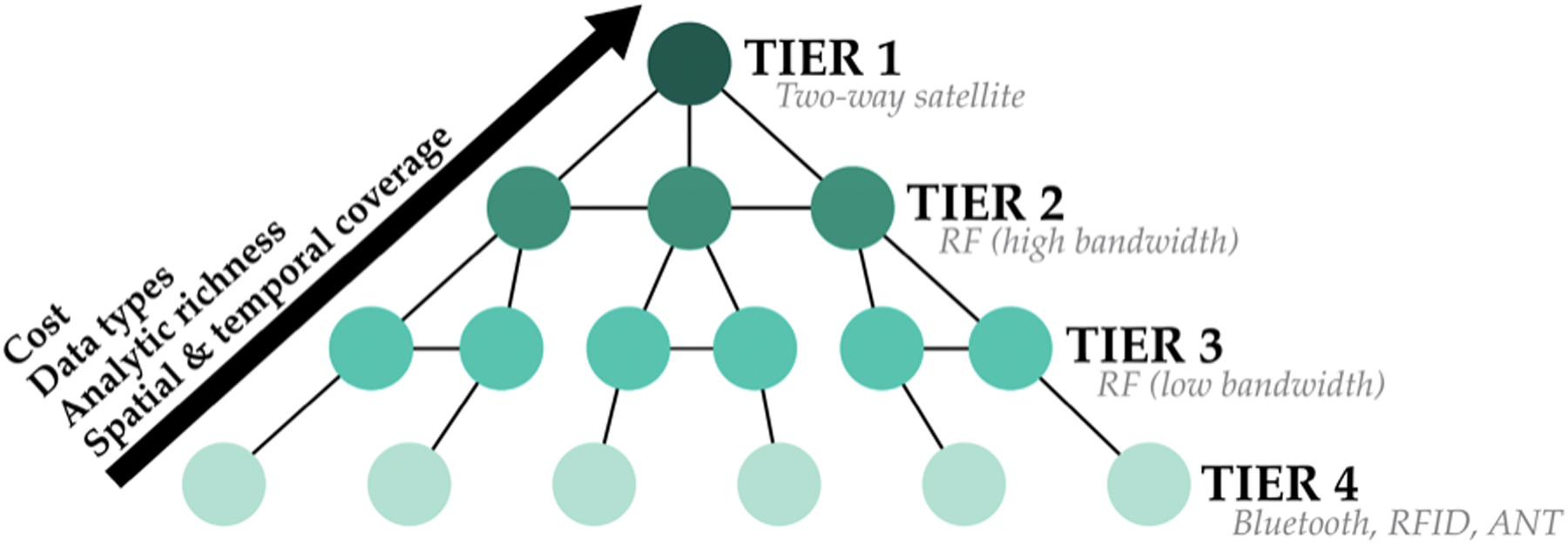
Conceptual model for hierarchical data collection, processing and communication in natural resources. In the lowest tier (Tier (4), data is collected from many resources and transmitted to the next tier (Tier (3) at very short ranges. At higher tiers (Tier 2 and (1), the number of resources decreases, but requisite bandwidth and range increases, as large amounts of data must be summarized and communicated across longer distances.

It is useful to consider a general model of how recently-available or improved positioning technologies and data topologies discussed in the early sections of this paper could be integrated to accommodate location-based services and big data themes in an IoT framework suitable for natural resource management in off-the-grid areas where data connectivity is otherwise limited. When reviewing technologies, it is evident that devices vary greatly in their capabilities, particularly with regard to bandwidth (data transfer capabilities), range, and accuracy. Although not explicitly addressed in this paper, these devices also tend to increase in cost as their bandwidth capacity increases. We propose a tiered model (Figure 15) in which low-bandwidth, low-cost, and short-range devices support functions locally in natural resources with smartphones serving as data hubs for individuals or equipment and data is exchanged among phones or tablets using miniaturized radios. Higher-order radios or devices with two-way satellite communications serve in upper levels of data transfer and processing, generally corresponding with higher levels of supervision and operational oversight. This model addresses and resolves important considerations for storing, processing and communicating big data through IoT to meet spatial and temporal considerations [14,16,220–222] in natural resources environments in a scalable way.

At the lowest level in positioning and communication capabilities, Bluetooth and related technologies (e.g., BLE, ANT) can share data in limited ranges, typically between 30 m and 200 m [188–199,227]. For many of these technologies, individual smartphones can serve as the hub for data collection, processing, and reporting; although in some cases, primary processing and generation of summary statistics may occur on wearable devices (e.g., an activity-tracking watch) or other sensors. In natural resources, these devices could collect and consolidate location or health data from the public, volunteers, or employees, such as wildland firefighters engaged on the fireline, loggers at individual timber harvesting operations, or a trail crew working in a remote wilderness area.

At the next higher level are miniaturized radios that pair with smartphones to form mesh networks, such as those from goTenna^™^, Beartooth^™^, and similar commercial providers. These devices are capable of data transfer over much greater distances, curbed only by the number of users, their spacing, and topographic impacts on line-of-sight. Although bandwidth is limited for these types of devices, they are particularly well suited for accumulating small data packet deliveries from many resources, or, in turn, delivering small bits of critical information broadly to large numbers of individuals. Thus, data accumulated by several smartphones in lower levels can be transmitted, for instance, to a single crew supervisor.

At one higher level are dedicated mesh data radios, such as those designed for military applications. Similar to miniaturized radios, these units have essentially unlimited, local range (e.g., greater than 25 km), depending on the number of users (nodes) in the mesh network; however, these purpose-built radios provide much greater bandwidth capabilities over those same ranges. Voice communication, video streaming, and other functions with higher data transfer rates are also possible. With higher bandwidths, these radios support a larger number of users transmitting small bits of data (e.g., user locations) at faster transmission rates, but the cost of individual units is generally five to ten times greater than the miniaturized radios.

At the top of the hierarchical data topology is two-way satellite communication. While satellite data transfer has the advantage of global coverage, and single user devices can function independently of others (e.g., without the need for mesh networking), it is also the most expensive option for transmitting data. Monthly subscription or per-usage fees compound quickly for multiple users; whereas data transfer through radio frequency is typically free in the United States after the purchase of a radio frequency license. Also, low data transfer frequencies (e.g., one user location every two minutes) make satellite-based solutions less desirable for occupational safety and health applications where real-time, local data sharing with co-workers in remote areas is more critical (e.g., Wempe et al. [21], Zimbelman et al. [23]) or where detection of precise equipment movements or equipment-worker interactions are required [22,86,87].

**Figure 16.**
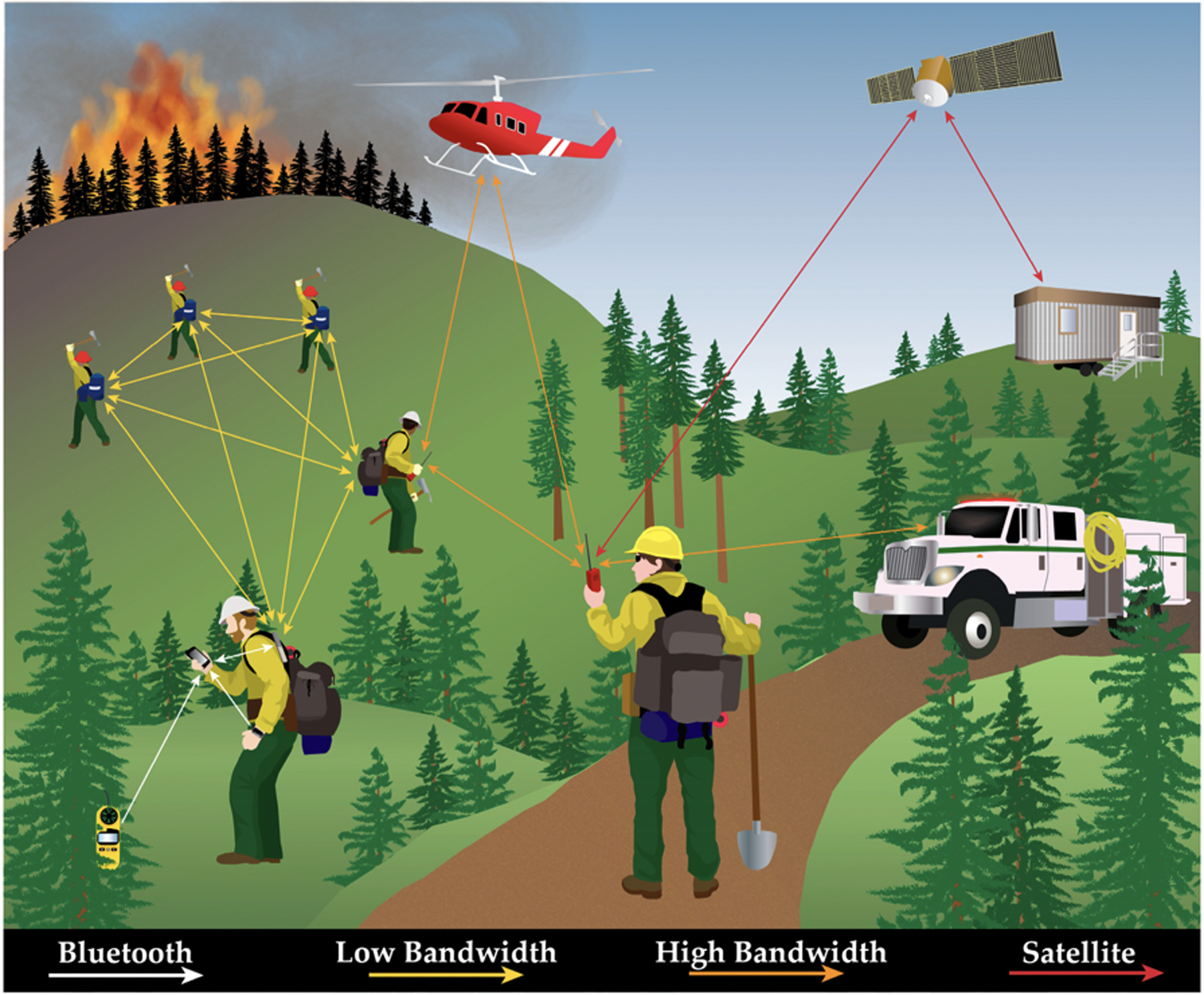
Hierarchical approach to location and data sharing in wildland firefighting. Smartphones serve as hubs for Bluetooth, BLE, or ANT devices such as an environmental sensor (bottom left) and wearable activity-monitoring devices on individuals. These data are processed locally on phones and summary data are transmitted via miniaturized radios such as goTenna, first by Bluetooth, BLE or ANT to the radio device, and then by radio frequency to other crew members. Crew bosses at a higher level of supervision receive data on advanced digital radios with higher bandwidth (and cost). For emergency alerts that require complete connectivity independent of a local network, and for the highest level of Incident Command, devices with two-way satellite data transfer are used.

Figure 16 illustrates an example of the hierarchy in a wildland fire application. In this scenario, miniaturized radios share the locations of resources (firefighters, engines, etc.) during initial attack operations on a large wildland fire at moderate transmission intervals among many users. These devices also deliver summaries of fire weather data detected by a Kestrel or similar device, transmitted to a single user’s smartphone via Bluetooth, and then broadcasted to other firefighters in the mesh network.

In the Figure 16 example, summary data quantifying movements or activities of groups of individuals, equipment, or localized weather conditions locally are then transmitted to a higher supervisory level or between multiple teams separated spatially on the landscape using a single, higher bandwidth digital radio. For example, if four 20-person hand crews and two 3-person engine crews are working on a fire, 86 paired smartphones might be accumulating and processing data from wearable sensors worn by all individuals. Individual-level summaries of worker health and work progress based on activity recognition modeling on wearable devices could be transmitted via 86 miniaturized radios to the six supervisors (four crew bosses and two engine bosses), each of whom have dedicated digital data radios with higher bandwidth capability. Crew-level summaries of these health, work progress, and local fire weather metrics could then be transmitted via the high-bandwidth data radios to other mid-level supervisors such as task force leaders, division supervisors, or others at higher levels. Satellite data transfer may be the preferred method for Incident Command (IC) exchanges, or possibly for lower level workers to communicate reliably with a regional or national command center in situations where a local radio network is not supported. It is also the primary option for communicating single locations to a distant (e.g., urban) public safety center in the event of medical emergencies or search and rescue (SAR).

Thus, raw sensor data from smartwatches and smartphones can be processed locally by those devices using coded models that summarize work activities in meaningful ways and reduce data storage and transfer size accordingly. The most critical data can then be transferred via data radios or other networks to devices that consolidate and summarize more complex spatial or temporal interactions among multiple users. In this way, the distributed system provides not only data transfer off-the-grid, but also calculations and computing power that are hierarchical, with the most critical summaries available for higher level managers. Additionally, depending on the type and urgency of transmitted data, there may be different processing and bandwidth needs for upstream vs. downstream data exchange. For example, infrequent (e.g., daily) downstream flow of critical alerts or updated imagery or maps from IC to lower-levels may take high priority at fixed times during the day, while upstream aggregation and distillation of field condition data from line workers upward.

This hierarchical model also applies to operational forestry and the wood supply chain, wildlife and fisheries management, ecology and ecosystem sciences, and other fields. In operational forestry, for example, data may be collected on logging equipment efficiency (e.g., wood production rate for each piece of equipment and associated stand conditions), current logging costs per hour, worker health and safety metrics, and the quantity and quality of products being processed in remote areas. This data may be summarized on smartphones and shared locally at the jobsite using miniaturized radios. As in the wildland fire example, data would then be transferred among operations foresters, wood procurement staff, or land managers using higher throughput radios. Critical summary information would ultimately be transmitted to large timberland owners, state or federal government agencies or sawmills in the top tier using high-bandwidth radios or satellite data communications. Feedback from the landowner or mill, such as product specifications, timing of operations, current product needs, or other information can be transmitted back to multiple jobsites with higher-level devices as well.

## 6. Emerging Research Needs

Technology for the use of real-time PNT in natural resources is evolving rapidly. As we develop new, mobile applications and data communications topologies to take advantage of improved real-time location and data sharing in forest operations, wildland fire, and more broadly in natural resources, several research needs emerge. Addressing these topics is necessary for moving from concept to practice and actualizing the potential benefits of PNT technologies, LBS, activity recognition, and IoT devices for analytics and decision support in natural resource management.

### 6.1. Development of Activity Recognition Models for Individual Worker and Equipment Tasks in Forestry, Wildland Firefighting, and Natural Resources

To further integrate activity recognition into natural resources, activity recognition models must be developed to characterize movements and conditions associated with various human, animal, and equipment work tasks. Activity profiles are being rapidly created for fitness and recreational activity monitoring, but applications quantifying work productivity in natural resources are limited. Becker et al. developed methodology and code to model swing movements of forestry machines [86], and Keefe et al. developed an activity recognition model for timber fallers [14]. However, further equipment and worker activity recognition detailing a wide range of forestry and wildland fire tasks are needed to characterize work activities in integrated, real-time analyses to improve productivity and efficiency and to reduce costs. Similarly, in fisheries and wildlife, development of individual-level activity data based on inertial sensors may enhance future research characterizing behavioral patterns in a variety of ways.

### 6.2. Development of New Sampling, Analytical, and Statistical Methods to Quantify Real-Time Resource Movements in Time and Space for Many Agents

Through our literature review process, it has been evident that GNSS-related research in natural resources has predominantly focused on accuracy of stationary GNSS devices or relatively simple movements and work processes of individual pieces of equipment or personnel [42,63]. An exception is Wempe and Keefe, in which the location and movements of equipment, ground workers, and multiple mobile jobsite hazards were quantified and summarized simultaneously at one second intervals using a GNSS-RF network [21]. By quantifying all components of logging, firefighting, or other systems simultaneously, there is tremendous potential to advance these fields and improve efficiency and safety through more sophisticated types of analytics. Informatics associated with real-time positioning and equipment- and personnel-based work activity recognition is both an exciting and intimidating field due to the sheer volume of data being generated and needing to be quantified. As an example, Keefe et al. showed that recording smartphone sensor data for just one work day for hand fallers generates approximately 118,000 rows of data when recorded at 10 Hz [14]. Consequently, quantifying both the movements and work activities of, for example, 500–1000 wildland firefighters and other support resources in real-time presents formidable challenges, particularly when data processing occurs on mobile devices without access to cloud computing.

In light of the diffusion of big data into IoT applications for natural resources, an important subject area for future research is the development and testing of methods to (a) transfer data efficiently in the hierarchical model, and (b) quantify forestry and firefighting operations in ways that reduce the dimensionality in time and space of data as it is collected and processed vertically (upstream) in the hierarchy. In addition to applied evaluation of functional data processing and communication systems in the field, there is also a need to address more basic analytical problems to support big data and IoT growth in natural resources. These include identification and evaluation of new, basic statistical methods to quantify complex point-process data moving in new patterns and at high resolution in time and space. Such methods must account for kinematic, positioning and timing data, including the time for software to process locations or other data, transmit that information to other devices, and then for receiving devices to process and interpret incoming data, often in a time-critical decision-support capacity. Further, since multiple sources of error interact on the landscape in complex ways, development of analytical approaches that either quantify the components of error in integrated systems or quantify net, cumulative error directly (e.g., Zimbelman et al. [22], Grayson et al. [85]), are needed.

Analysis techniques for spatiotemporal data structures with many resources moving simultaneously may be applied to a variety of fields, both within natural resources and beyond (e.g., human behavior research, safety, and defense). For example, one might consider automated recognition of troop movement patterns in military environments, or the simultaneous movements of soccer players on a field. In each example, careful consideration of research and management goals early on can help to optimize efficient sampling frequency and network design for upstream collection, processing and consolidation of information in time and space.

### 6.3. Evaluation of Data Network Quality in Mission-Critical Operations with many Resources

Much of the applied research we have referenced on data sharing in forestry, including Wempe and Keefe [21], Zimbelman and Keefe [23], and Becker et al. [86], has resulted from small, controlled experiments, or sampling with a small number of networked location-sharing devices (typically less than ten). However, to effectively deploy data networks in mission-critical wildland fire, disaster response, and other public safety applications in remote areas, testing technologies on a larger scale with more resources is imperative. The behavior of ad hoc networks when hundreds or even thousands of resources are being monitored is likely to generate a variety of bandwidth and connectivity issues. Further research is needed to evaluate the potential use of unique frequencies for different component systems sharing data simultaneously at low-level nodes in the hierarchical network as well as the slowing and failure of data transfer as the number of users increases. There are many, potentially useful applications for sharing activity recognition model predictions among phones and wearable sensors, particularly in the context of work productivity and worker health monitoring to improve safety. However, ad hoc networks may become clogged with this data when they are most needed, namely, on large incidents where conditions are changing quickly, and the rapid transfer of information among many agents is most critical. For this reason, it may be useful for mesh networks to establish enhanced emergency override controls that throttle non-critical communications, prioritizing top-level uses as the system becomes impaired.

### 6.4. Development of Integrated Formats and Protocols for Sharing Augmented PNT and Other Big Data

When GNSS-derived positions are modified in some way, such as through augmentation or integration with GNSS-INS localization, some retention and transfer of those changes is needed for quality assurance and quality control in downstream applications, or subsequent meta-analyses. Thus, development of integrated methods, protocols, and standards for transferring this modified location data among networked devices is needed. As points are shared on an ad hoc network, the timestamp of the original GNSS fix may be carried through the network to other devices, but temporal referencing critical for subsequent applications may become lost or unclear depending on the programming logic determining how different devices and mobile apps transfer and consolidate data. This could occur for example, when the position and vector heading of a forest machine is not sent to another machine due to a missed or delayed radio signal, which could result in navigational errors. Correcting potential errors of this sort may be as simple as adding additional fields or data columns that carry subsequent timestamps associated with when information is sent or received each time data is transferred between devices. Thus, the development of best management practices or standardized protocols for sharing location data among IoT devices that may be lagged in time is important for future research. Similarly, development of consistent formats by which supplementary data may be transferred among devices along with position information will facilitate rapid and consistent growth of enterprise applications. This is particularly important for public safety, in which the well-being and lives of first responders depend on accurate PNT data. Given the variety of technologies and correction methods that may ultimately be applied to improve positioning accuracy in forested environments, it may also be most useful to develop methods or protocols for providing observed end-user accuracy estimates when possible. In wildlife and fisheries research, the sharing and of big data, and thus availability for higher tier meta-analysis and comparative studies, may be affected by whether researchers tend toward collaborative and altruistic behavior, participate in telemetry networks, and other factors [228].

### 6.5. Landscape-Scale Mapping of Vegetation and Canopy Impacts on Position Accuracy

Forest vegetation has repeatedly been shown to markedly diminish GNSS positioning accuracy due to multipath errors [48,229–234]. Vegetation and topography also degrade local transmissions sent by Bluetooth or other radio-based technologies [235]. To advance the integration of real-time positioning systems for critical operations in natural resources, new approaches to quantify the impacts of vegetation and topography systematically over large landscapes are necessary. For instance, baseline, multipath errors for GNSS-based positioning and RSSI loss for conventional frequencies (VHF, UHF, 900 MHz) could be mapped across expansive areas using remote sensing satellite or airborne LiDAR data. These predicted errors could then be integrated into end-user PNT applications to aid mission planning for operational and tactical support.

### 6.6. Developing Applications to Improve Worker and Recreational Health and Safety in Natural Resources

Concurrent with the development of activity recognition models to quantify work described in Section 6.1, new, smart methods for quantifying worker health are needed to help monitor and improve health and safety of individuals employed in natural resources. For example, wearable or mobile devices could quantify heat stress, over-exertion, and related factors during wildland firefighting activities. Smart, person-down alerts for logging contractors could improve incident detection and response [88], and similar methods could be developed to create overboard alerts for fisheries workers. Also, methods to detect drowsiness and reduced awareness during equipment operation or other high-risk tasks may help to reduce incident-related injuries. Numerous smartphone-based health apps for achieving health and fitness goals and monitoring long-term conditions already exist [236–238]; tailoring these kinds of applications to natural resources work and recreational activities can help to improve wellness for forest workers.

### 6.7. Evaluating Potential Adverse Health Impacts of Wearable Technology Use

As smartphones, smartwatches, and the numerous technologies discussed throughout this paper become omnipresent in natural resources because of the many beneficial applications, a consideration for managers and researchers is whether there may be potential adverse health impacts of regularly using wearable and carried electronic devices, with increasing human exposure over time. While most prior research indicates minimal health impacts associated with RF exposure, blue light associated with smartphone, tablet and laptop screens has received prior attention related to possible health effects on sleep [239]. Millimeter wave wireless impacts on the body have been identified as an area for future research [240]. Smartphone addiction is a problem receiving attention in recent research as well [241] and has been associated with anxiety and depression [242]. Additionally, repetitive use of smartphones may be associated with increased upper extremity pain [243]. While documented impacts of device use appear to be relatively minor, perception of possible adverse health impacts of increasing device use is a common concern raised when discussing related research and applications.

### 6.8. Addressing and Establishing Policies to Resolve Social and Ethical Concerns Associated with Sharing Worker Health Data

Regardless of any possible direct health impacts discussed in the previous section, the sharing of personal activity monitoring data and associated personal health metrics collected by wearable sensors introduces a range of important questions about ethical propriety and social acceptance [244–247]. Before these data are widely integrated into analytics for natural resources, research must first apprehend worker perspectives on the sharing of personal data, potentially with supervisors. Despite having a variety of well-intentioned wellness and safety benefits for employees and recreationists, the use of personal, identifiable information will require various protocols, such as those for ensuring users’ voluntary consent. As research and development in LBS, IoT, activity recognition, and other sensor-based applications push the limits of privacy with emerging big data analysis, new challenges and questions emerge for natural resource management. For example, what methods are there for providing anonymity to individual workers while monitoring work crews’ overall wellness in aggregate? What are the responsibilities of employers who have access to health data, as is becoming increasingly common, to report apparent health anomalies or concerns? These are complex issues for natural resources managers and researchers alike to grapple with, and policy research evaluating options and solutions will be needed. As mobile devices continue to become the hub for a variety of local data connectivity linking internal and external sensors, another important consideration is evaluating whether employees are willing to use their smartphones for work-related applications.

### 6.9. Normative Research Evaluating New Use Cases of LBS and IoT Concepts to Improve Natural Resource Science and Management

In urban environments, the use of LBS in marketing and sales is growing rapidly. Retailers can tailor shopping experiences to the consumer, identifying individuals through their smartphones using Bluetooth or WiFi [26,227,248]. Bluetooth beacons placed under displays or within objects in the store are used to adapt shopping and sales experiences to individuals and their preferences based on available information such as purchase history or internet searches, much as the advertisements displayed on websites are personalized based on browsing history. In what ways can we take advantage of a similar level of human physical interaction with objects, equipment and the environment in forestry and wildland fire? For example, can similar Bluetooth-enabled beacons, RFID or NFC tags guide equipment operators to move optimally between sorted log decks on active timber sales with information that carries through to sawmill operations? Can low-cost beacons be used along with conventional ribbon flagging to identify hazard trees or other safety concerns and then alert firefighters and loggers to their presence based on smartphone LBS? IoT sensors that monitor tree growth are now becoming available; in the future, can log product types within the area of interest (AOI) be reported to equipment operators, so they can preferentially harvest or bunch logs of a particular product in sequences that simplify downstream log processing and loading activities? How can we use technology in ways that increase efficiency across forest inventory, precision silviculture, harvesting equipment automation, and wildland fire detection and response? At broader scales across natural resource disciplines, how can we integrate IoT concepts across forest, fire, wildlife and fisheries management to improve efficiencies in monitoring of multiple resources and generate synergistic data to better manage resources cost effectively and with newly integrated data structures?

## 7. Conclusions

We have provided a review of available PNT technologies in forested environments, and for several emerging technologies and concepts that are likely to impact natural resource management. Specifically, we reviewed PNT technologies including GNSS, Bluetooth, UWB, RFID, and QR codes. We also reviewed concepts that may influence natural resources in the future, including LBS, geofences, wearable technology, activity recognition, mesh networking, the IoT, and big data. It is clear from our review that new possibilities for monitoring, analyzing, and reporting the movements of people, equipment, wildlife and objects in remote areas are emerging quickly and will advance safety, work productivity, and research methods in ways that have not previously been feasible.

Currently, there are relatively few examples of applying IoT, LBS, and big data to operational forestry and wildland fire, outside of the common use of remote sensing and fire behavior modeling. In the future, however, we will likely see a much wider range of big data and sensor-based topologies and concepts diffusing from computer science, marketing, and other fields. Solutions for sharing data in remote areas will generally include a mix of short-range (Bluetooth, BLE, ANT), radio, cellular, and satellite technologies, but will require some modification of currently available applications designed to function seamlessly in urban areas. For this reason, the off-the-grid approaches being developed for day-to-day data sharing in natural resources are also of interest and importance for supporting public safety in urban areas during environmental disasters (hurricanes, floods, and fires), military conflicts, and other situations when cellular and internet communication may be denied or degraded. Emerging, map-based positioning approaches such as SLAM and map matching, perhaps when combined with elements of inertial navigation or LBS, are likely to increase in importance.

To best take advantage of the new, big data paradigm in natural resources, we have proposed a simple hierarchical model for location and data sharing in IoT applications off-the-grid. At lower tiers with many resources (e.g., hundreds of people and equipment), sensor data is processed and transmitted locally with low-cost, low-bandwidth devices. At higher tiers, aggregated data is transferred over longer distances via higher-cost, higher-bandwidth communications equipment, with the most critical data sent through global networks (e.g., satellites). This tiered structure corresponds closely with Incident Command on wildland fires, the general organization of the forest products supply chain in operational forestry, wildlife population monitoring, and many other federal, state, and industrial organizational structures in natural resources.

To best support the continuing emergence of IoT and LBS concepts into big data analysis for natural resources, future research should focus on (1) developing activity recognition models for common forestry and firefighting work activities, so wearable watches and phones can quantify worker safety, health, and productivity in real time; (2) developing and evaluating new basic and applied statistical and analytical methods accounting for the movement of many resources and their interactions with one another and their surrounding environment; (3) assessing the applied functionality of processing and sharing data in networks with many resources working in forested and perhaps smoky environments; (4) developing integrated methods, protocols, and standards for transferring modified location data; (5) quantifying the impacts of vegetation and topography on position accuracy over large landscapes; (6) developing applications to improve worker and recreational health and safety in natural resources; (7) evaluating potential adverse health or safety impacts of smartphone and wearable technology use; (8) addressing social and ethical concerns associated with sharing worker personal data; and (9) developing normative approaches to evaluate new uses of LBS and IoT concepts based on interactions of smartphones and physical objects in forested and other remote environments to improve natural resource science and management.

## Figures and Tables

**Table 1.** PNT technology spatial range and accuracy.

Method	Range	Static Position Accuracy^1^	Technology	Reference
*Single User*				
GNSS	Global	1–2 m (5–10 m canopy)	Recreational & mapping grade	[29]
RTK-GNSS	Global	5 cm (1 m canopy)	Survey grade	[29]
PPP-GNSS	Global	< 5 cm (> 0.5 m canopy)	Survey grade	[67,70]
GNSS-INS	Global	GNSS/INS: 0.5–1 m PPP/INS: 5–10 cm	Tightly-coupled systems^2^	[103,104,105,106]
*Multi-node* ^3^				
GNSS-RF	Line-of-sight	2–4 m (< 10 m canopy)	Recreational grade – U.S. GPS only	[21]
UWB	100 m (15 m NLOS)	3 cm-0.5 m (1 m NLOS)	Commercial grade	[77,95,96]
Bluetooth	up to 50 m up to 100 m (20 m indoors) up to 200 m (40 m indoors)	2–5 m (BLE, indoor)	BLE Bluetooth 4.x Bluetooth 5.0	[117,118]
RFID	Up to 1 km	< 20 cm-5 m	Active UHF RFID (RSS)	[77,126,127,128,129]
QR code	Global	Same as GNSS	GNSS	[29]

1 Root mean square error (RMSE) for all values except Bluetooth and RFID, which are reported as mean error;

2 Raw GNSS measurements are used to aid INS;

3 Refers to whether positions are readily transferred to other devices (single user solutions can easily become ‘multi-node’ if integrated with GNSS-RF, Bluetooth, etc.).

## Appendix A

Documentation of the search methodology, inclusion criteria, and related details following PRISMA guidelines are required for publication of literature reviews in *Forests*. In this Appendix, we describe characteristics of our search methodology that did not seem to flow well within the storyline in the manuscript and seemed better incorporated here.

### Appendix A.1 Search Methodology

We used Google Scholar as our principle database for reviewing literature. Google Scholar includes more non-peer-reviewed gray literature than other databases such as Web of Science, but is also faster at indexing and returning citations for recently published literature. For example, the lead author of this manuscript has approximately 200% of the citations from published peer-reviewed manuscripts at the time of writing this article for papers published between 2013 and 2017. Thus, given that we were reviewing the state of the art in a rapidly changing subject area, effort and time were allocated to the Google Scholar database as the preferred source. In addition to results returned from the database, papers that the co-authors were already familiar with but may not necessarily have shown up in database results were also included.

Table A1 shows search terms related to positioning technologies for forestry and wildland fire applications, associated with Section 2 of the manuscript.

**Table A1.** Search terms used in Google Scholar for manuscript Section 2.

Manuscript Subject	Search Term 1	Search Term 2	Search Term 3
GPS (forestry)	GPS forestry	GPS forest accuracy	GPS forest range
GPS (wildland fire)	GPS wildland fire	GPS wildland fire accuracy	GPS wildland fire range
GNSS (forestry)	GNSS forestry	GNSS forest accuracy	GNSS forest range
GNSS (wildland fire)	GNSS wildland fire	GNSS wildland fire accuracy	GNSS wildland fire range
GNSS-RF (forestry)	GNSS-RF forestry	GNSS-RF forest accuracy	GNSS-RF forest range
GNSS-RF (wildland fire)	GNSS-RF wildland fire	GNSS-RF wildland fire accuracy	GNSS-RF wildland fire range
Bluetooth (forestry)	Bluetooth forestry	Bluetooth forest accuracy	Bluetooth forest range
Bluetooth (wildland fire)	Bluetooth wildland fire	Bluetooth wildland fire accuracy	Bluetooth wildland fire range
Ultra wideband (forestry)	Ultra wideband forestry	Ultra wideband forest accuracy	Ultra wideband forest range
Ultra wideband (wildland fire)	Ultra wideband wildland fire	Ultra wideband wildland fire accuracy	Ultra wideband wildland fire range
INS (forestry)	Inertial navigation system forestry	Inertial navigation system forest accuracy	Inertial navigation system forest range
INS (wildland fire)	Inertial navigation system wildland fire	Inertial navigation system wildland fire accuracy	Inertial navigation system wildland fire range
RFID (forestry)	RFID forestry	RFID forest accuracy	RFID forest range
RFID (wildland fire)	RFID wildland fire	RFID wildland fire accuracy	RFID wildland fire range
QR code (forestry)	QR code forestry	QR code forest accuracy	QR code forest range
QR code (wildland fire)	QR code wildland fire	QR code wildland fire accuracy	QR code wildland fire range

The terms in Table A2 were searched to define and illustrate broader, emerging data science concepts, technologies, and trends related to positioning and other sensor-based data topics both generally and in the purview of forestry.

**Table A2.** Search terms used in Google Scholar for manuscript Section 4.

Manuscript Sub-section	Search Term 1	Search Term 2	Search Term 3
4.1.	Location-based services	Location-based services forest	Location-based services forestry^1^
4.2.	Geofences	Geofence forest	Geofence forestry
4.3.	Wearable technology	Wearable technology forest^1^	Wearable technology forestry
4.4.	Activity recognition	Activity recognition forest^1^	Activity recognition forestry^1^
4.5.	Mesh networking	Mesh network forest	Mesh network forestry
		Wireless sensor network forest	Wireless sensor network forestry
4.6.	Internet of Things	Internet of Things forest^1^	Internet of Things forestry
4.7.	Big data	Big data forest^1^	Big data forestry

1 No relevant results in first two pages of search (ten results per page).

### Appendix A.2 Screening, Inclusion, and Bias

For all results returned from Table A1 search terms, abstracts were screened to determine their relevance to the core theme of the manuscript using the following criteria: (1) does the article generally include information relevant to emerging frontiers in real-time positioning for operational forestry and wildland fire? and (2) does the article include information relevant to the particular technology being studied (e.g., GNSS-RF)? In addition to results from the literature searches, additional information was included on technology and systems available. For example, there were relatively few literature sources on integrated wearable and positioning systems, yet there were a number of new technologies being actively marketed for use in forestry and wildland fire as of 2018. Additionally, because our manuscript was intended to serve in part as a reference document on positioning technologies, illustrative figures were created to highlight key characteristics of important positioning technologies or IoT concepts, as they may relate to forestry. For example, Figure 9, Figure 10, and Figure 11 do not explicitly represent published papers, but show technology concepts as they might be applied to natural resource management.

Abstracts displayed for Table A2 Search Term 1 were screened based on the following criteria: (1) does the article define or contribute to the basic understanding of the concept (e.g., location-based services)? or (2) does the article describe applications of the concept? For Search Terms 2 and 3, abstracts were reviewed for reference to the core concept and relevance to forestry or wildland fire.

It is required for PRIMSA documentation to note bias that may be present in systematic reviews. As noted in the Abstract and Introduction, this manuscript includes two distinct systematic reviews, but then also includes supplemental literature from fisheries and wildlife, on video object identification and relative positioning, and selected other terms and concepts. Thus, the inclusion of additional terms and concepts beyond the original systematic searches is a form of bias. We felt it best served the spirit of the review to cover as much related literature as possible and that these contributed to the body of knowledge for interested readers. Additionally, bias in subject areas was introduced by the authors insofar as we supplemented literature identified in systematic reviews with known references in some cases.
